# Evaluation of Dose-Dependent Obesity and Diabetes-Related Complications of Water Chestnut (Fruit of *Trapa japonica*) Extracts in Type II Obese Diabetic Mice Induced by 45% Kcal High-Fat Diet

**DOI:** 10.3390/medicina58020189

**Published:** 2022-01-26

**Authors:** Hyun-Gu Kang, Khawaja Muhammad Imran Bashir, Ki-Young Kim, Su Shin, Min-Woo Choi, Eun-Jin Hong, Seong-Hun Choi, Joo-Wan Kim, Jae-Suk Choi, Sae-Kwang Ku

**Affiliations:** 1 Department of Anatomy and Histology, College of Korean Medicine, Daegu Haany University, Gyeongsan 38610, Korea; ggg5626@naver.com (H.-G.K.); ck0190@hanmail.net (S.-H.C.); 2 German Engineering Research and Development Center for Life Science Technologies in Medicine and Environment, 31, Gwahaksandan 1-ro, 60 bean-gil, Gangseo-gu, Busan 46742, Korea; imranagrarian3@gmail.com; 3 Research Institute, Bio Port Korea Inc. #207, 7, Hoenggye-gil, Ilgwang-myeon, Gijang-gun, Busan 46048, Korea; kyk1967@bioportkorea.com (K.-Y.K.); sshin@bioportkorea.com (S.S.); mwchoi@bioportkorea.com (M.-W.C.); ejhong@bioportkorea.com (E.-J.H.); 4 Gyeongnam Veterinary Service Laboratory, 104, Chojeonbuk-ro, Jinju 52733, Korea; warii@hanmail.net; 5 Department of Food Biotechnology, College of Medical and Life Sciences, Silla University, 140, Baegyang-daero 700 beon-gil, Sasang-gu, Busan 46958, Korea

**Keywords:** diabetes, obesity, *Trapa japonica*, type II obese diabetic mice, water chestnut

## Abstract

*Background and Objectives*: The currently used pharmacological agents for metabolic disorders such as type II diabetes have several limitations and adverse effects; thus, there is a need for alternative therapeutic drugs and health functional foods. *Materials and Methods*: This study investigated the pharmacological effects of water chestnut (fruit of *Trapa japonica*) extracts (WC: 50–200 mg/kg) for type II diabetes using a 45% Kcal high-fat diet (HFD)-fed type II obese diabetic mice model for a period of 84 days, and the effects were compared to those of metformin (250 mg/kg). *Results*: Increases in body weight, serum biochemical indices such as triglycerides, low-density lipoprotein, and blood urea nitrogen, increases in antioxidant defense system enzymes such as catalase, superoxide dismutase, and glutathione, and mRNA expressions (such as AMPKα1 and AMPKα2) in the liver tissue and mRNA expressions (such as AMPKα2 mRNA, leptin, and C/EBPα) in the adipose tissue were observed in the HFD control group. The WC (50 mg/kg)-administered group showed no significant improvements in diabetic complications. However, HFD-induced obesity and diabetes-related complications such as hyperlipidemia, diabetic nephropathy, nonalcoholic fatty liver disease (NAFLD), oxidative stress, activity of antioxidant defense systems, and gene expressions were significantly and dose-dependently inhibited and/or normalized by oral administration of WC (100 mg/kg and 200 mg/kg), particularly at a dose of 100 mg/kg. *Conclusions*: The results of this study suggest that WC at an appropriate dose could be used to develop an effective therapeutic drug or functional food for type II diabetes and various associated complications, including NAFLD.

## 1. Introduction

Metabolic syndrome, a cluster of dyslipidemia, central obesity, hypertension, and/or insulin resistance, is associated with increased risk of type 2 diabetes [1,2]. When a person is obese, adipocytes not only store lipids as a long-term energy source but also secrete various adipokines, which are involved in metabolism and inflammation of adipocytes and non-adipocytes, causing chronic inflammation and leading to related metabolic complications such as insulin resistance [3]. In recent years, the incidence of the metabolic syndrome known as Type II diabetes has been increasing worldwide due to obesity caused by lack of exercise along with a high-calorie diet [4]. The number of people with diabetes rose from 108 million in 1980 to 422 million in 2014 [5]. In 2014, 8.5% of adults aged 18 years and older had diabetes. In 2019, diabetes was the direct cause of 1.5 million deaths, and 48% of all deaths due to diabetes occurred before the age of 70 years. Between 2000 and 2016, there was a 5% increase in premature mortality rates (i.e., before the age of 70) from diabetes. Prevalence has been rising more rapidly in low- and middle-income countries than in high-income countries. More than 95% of people with diabetes have type 2 diabetes [5].

Excessive fatty acids intake induces triglyceride (TG) accumulation in various tissues and insulin resistance in adipocytes. This causes an increase in fatty acid absorption by non-adipocytes due to excess transport proteins and fatty acid binding, adversely affecting the insulin-mediated glucose metabolism. Meanwhile, in the pancreas, prolonged exposure to free fatty acids leads to a recurring vicious cycle in which insulin secretion is impaired by the lipotoxicity mechanism [6]. This leads to a high accumulation of free fatty acids in the liver, resulting in insulin resistance and release of a large amount of glucose from the liver [7]. The TG accumulation in hepatocytes and fat accumulation in the liver causes nonalcoholic fatty liver disease (NAFLD), which results in fibrosis due to necrosis of hepatocytes [8]. Therefore, the balance of fat synthesis and degradation in hepatocytes has been an important therapeutic target that can inhibit the induction of insulin resistance and NAFLD caused by metabolic syndrome [9].

Severe obesity is induced in rodent mice by supplying a high-fat diet (HFD). Compared to normal pellet diet-fed (NFD) mice, remarkable hyperglycemia, insulin resistance, nonalcoholic steatohepatitis (NASH), mild diabetic nephropathy, and hyperlipidemia were recognized in HFD-induced obese mice [10,11,12,13]. This shows that HFD supply induces diabetic obesity similar to human metabolic syndrome in ICR mice. Various experimental animal models have been used for testing metabolic-related therapeutic agents, but most of those cause severe obesity and/or hyperglycemia, and are inadequate for the development of preventive or functional foods [14]. However, the experimental animal model using HFD is known to cause obesity and hyperglycemia in an appropriate degree for efficacy evaluation of the preventive and functional foods for metabolic syndromes, including NAFLD [12,13,15]. Therefore, this study was conducted using a HFD (45% Kcal) mouse model to evaluate the pharmacological activity of water chestnut (WC) for metabolic syndrome including obesity in a dose-dependent manner. The HFD supply period was set to 13 weeks, including one-week HFD adaptation period, which can sufficiently induce metabolic syndrome based on the efficacy evaluation in the previous HFD mouse models [11,12,13,15].

Due to several limitations associated with the use of currently available pharmacological agents for metabolic syndrome, such as adverse effects and high rates of secondary failure [16], healthcare professions and patients are considering complementary and alternative approaches with low side effects and higher effectivity [13,15]. Most recently, appropriate control of oxidative stress, which causes diabetes and related complications, has emerged as the most important method for diabetes treatment [12,13,15,17]. Furthermore, development of α-glucosidase blockers or antioxidants with low side effects and higher effectiveness has been attempted [12,13,18,19]. Among these, metformin is well known as an AMPK activator as a representative oral biguanide antidiabetic treatment [20,21], and it is the first drug used to treat patients with overweight and type II diabetes with normal kidney function [22]. In this experiment, on the basis of previous medicine efficacy experiments [12,13,15], metformin (250 mg/kg) was used as a positive control drug, and comparative evaluation was performed.

*Trapa japonica* Flerov., belonging to the genus *Trapaceae* [23], grows in shallow water fields, ponds, or reservoirs in Asia and Europe [24], and its fruit is known as water chestnut [25]. *T. japonica* has been used as an ethno-medicine for the treatment of hangover after alcohol consumption, gastric ulcer, dysentery, and diarrhea [26]. It contains dietary fibers, high starch levels, and polyphenols such as ellagic acid, trapain, gallic acid, and eugeniin [27,28]. Previously *T. japonica* has exhibited anti-adipogenic [29,30], antidiabetic [25,31], antioxidant, and anti-inflammatory activities [24,26,31].

The present study intended to confirm the dose-dependent beneficial potential of WC on mild diabetic mice (the 45% Kcal HFD-fed mice) compared to those supplied metformin (250 mg/kg), as a representative antidiabetic drug for type II diabetes [32,33].

## 2. Materials and Methods

### 2.1. Test Material

Water chestnut (WC; fruit of *Trapa japonica* Flerov.) extract was provided by Bioport Korea INC (Yangsan, Korea). The WC extract is a light brown powder that well dissolves up to 20 mg/mL in distilled water. The WC extract was kept at −20 °C until further use.

### 2.2. HPLC Analyses

The amount of ellagic acid in WC extract was quantified using Agilent HPLC 1200 (Agilent Technologies, Inc., Santa Clara, CA, USA) equipped with a Variable Wavelength Detector (G1314B, Agilent Technologies, Inc.) and Capcell Pak C18 UG120 column (4.6 mm × 250 mm, 5 μm; Osaka Soda Co. Ltd., Osaka, Japan). The WC extract and standard ellagic acid were dissolved by methanol (MeOH). The filtrates for HPLC analysis were obtained through 0.45 μm membrane filter. The column was kept at 40 °C during analysis, and ellagic acid was analyzed at 254 nm. A mobile phase consisted of a mixture of 0.03% phosphoric acid and acetonitrile (84:16) [25]. Standard ellagic acid was purchased from Sigma-Aldrich LLC. (#BCBZ2951; St. Louis, MO, USA). The 20 μL of each sample was injected at a flow rate of 1.0 mL/min, and the results were qualitatively analyzed.

### 2.3. Experimental Animals

A total of 60 six-week-old female SPF/VAF CrljOri:CD1(ICR) mice (OrientBio, Seungnam, Korea) were acclimated to the laboratory environment for seven days and again acclimated to high-fat diet (HFD) for 7 days. Four to five animals per transparent plastic cage were allocated to a humidity (40–45%) and temperature (20–25 °C) controlled room. Light was provided for 12 h, and standard rodent chow (Purinafeed, Seungnam, Korea) and tap water were provided freely [34].

The experimental animals (a total of six groups containing ten animals per group) were allotted as follows:

1. Normal control mice (NFD-administered group);
2. HFD control mice (HFD-administered group);
3. HFD supply control group (metformin—250 mg/kg group);
4. WC high dose (200 mg/kg), HFD-supplied experimental group;
5. WC intermediate dose (100 mg/kg), HFD-supplied experimental group;
6. WC low dose (50 mg/kg), HFD-supplied experimental group.

The humane endpoints were established in this study according to the results of Morton and Griffiths [35].

### 2.4. Selection of the Test Substance Dosage

In this experiment, the highest dose of WC was evaluated in the previous experimental animal model [25,26,31]. The medium and low doses were set at 100 mg/kg and 50 mg/kg with common ratio 2, respectively. By dissolving WC in sterile distilled water, 10 mL/kg was administered [36,37]. In addition, the dose of metformin (metformin hydrochloride; Wako Pure Chemical Industries, Ltd., Osaka, Japan) used as a reference drug was set at 250 mg/kg based on the previous studies [12,13,15].

HFD, 45% Kcal HFD (Table 1; Research Diet, New Brunswick, NJ, USA; Cat. No. D12451), was supplied to mice starting from one week before the test substance administration during the entire experimental period. In the normal control group, general NFD (Table 1; Purinafeed, Seungnam, Korea; Cat. No. 38057) was supplied instead of HFD.

### 2.5. Administration of the Test Substance

The WC (20, 10, and 5 mg/mL) and metformin (25 mg/mL) were dissolved in distilled water and administered orally once daily for 84 days at a dose of 10 mL/kg (final concentration of WC: 200, 100, and 50 mg/kg; final concentration of metformin: 250 mg/kg) using a 1 mL syringe attached with a metal sonde [36,37]. Before test substance administration, the mice were immobilized by holding the skin and tail of the neck and backbone with one hand. To apply the same administration and restrain stresses in the normal and HFD control groups, only sterile distilled water instead of the test substance was forcibly administered orally for the same period.

### 2.6. Changes in Body Weight

Changes in body weight were recorded at the eighth day (immediately before starting HFD supply), one day before starting administration, at initial supply day, and then on weekly intervals till the end of experiment using an automatic electronic balance (Precisa Instrument, Dietikon, Switzerland). To reduce the feeding differences, at initiation and at termination of test substance administration, all experimental animals were fasted overnight (no water provision for at least 12 h). Additionally, body weight gains were also calculated during the acclimation period (from day eight to day zero of test substance administration) and during the administration period (from day zero to day 84 of test substance administration) using Equations (1) and (2).

Body weight gains (g) during acclimation period = Body weights at initiation of administration − body weights at initiation of HFD supply(1)

Body weight gains (g) during administration period = Body weight at termination − body weights at initiation of test material administration(2)

### 2.7. Measurement of Body Fat Density: Abdominal and Total Fat Mass (%)

The mean fat densities in the abdominal cavity regions and the total body of each mouse were analyzed using in live DEXA (InAlyzer; Medikors, Seungnam, Korea), at the end of the experiment with continuous supply of test substance for 84 days [11,12,13,15].

### 2.8. Measurement of Blood Glucose Level

At the end of the experiment with continuous supply of test substance for 84 days, blood from vena cava of each mouse was collected under inhalation anesthesia with 3% isoflurane for induction and 1.5–1.8% isoflurane for maintenance of anesthesia (in the mixture of 28.5% O_2_ and 70% N_2_O; Hana Pharm. Co., Hwasung, Korea) using rodent inhalation anesthesia apparatus (Surgivet, Waukesha, WI, USA) and rodent ventilator (Harvard Apparatus, Cambridge, UK). The blood samples were stored in NaF glucose vacuum tubes (Becton Dickinson, Franklin Lakes, NJ, USA), and plasma was separated and stored in an ultra-deep freezer (Sanyo, Tokyo, Japan) at −150 °C until further analysis. An automated blood analyzer (Fuji Medical System Co., Ltd., Tokyo, Japan) was used to measure blood glucose levels.

### 2.9. Serum Biochemical Analyses

Serum from vena cava blood samples was separated by centrifugation at 15,000 rpm for 10 min under room temperature, and serum AST, ALT, ALP, LDH, GGT, BUN, creatinine, total cholesterol, and TG levels were analyzed with automated blood analyzer (Fuji Medical System Co., Ltd., Tokyo, Japan).

Serum insulin and blood HbA1c levels were measured by a mouse insulin ELISA kit (Alpco Diagnostics, Windham, NH, USA) and an automate HbA1c measuring equipment (Infopia, Anyang, Korea), respectively [11,12,13,15].

### 2.10. Organ Weight Measurements

At sacrifice, the weights of pancreas, liver, left kidney, abdominal wall-deposited fat pads, and left periovarian fat pads were recorded in gram levels, and to reduce the differences from individual body weights, the relative weights (% of body weights) were also calculated using absolute weight and body weight at sacrifice by following Equation (3) as demonstrated in our previous reports [11,12,13,15], with some modifications.

Relative organ weights (%) = (Absolute organ weights/Body weight at sacrifice) × 100(3)

### 2.11. Lipid Composition Measurement in Feces

Lipids were extracted from feces collected 8 h after administration of the last test substance [38]. The fecal TG and total cholesterol levels were recorded using a commercial total cholesterol colorimetric assay kit (Cayman, Ann Arbor, MI, USA) [11,12,13,15].

### 2.12. Measurement of Liver Lipid Peroxidation and Changes in Antioxidant Defense Systems

The GSH and MDA contents, and SOD and CAT enzyme activities in mouse hepatic tissues were analyzed. Weighed separated hepatic tissues were homogenized in ice-cold 0.01M Tris-HCl by bead beater (TacoTMPre, GeneResearch Biotechnology Corp., Taichung, Taiwan) and ultrasonic cell disruptor (Madell Technology Corp., Ontario, CA, USA), centrifuged at 12,000× *g* for 15 min [39], and stored in an ultra-deep freezer at −150 °C until further use.

The liver lipid peroxidation levels were analyzed by determining the MDA using thiobarbituric acid test [40]. Total protein content was determined by Lowry et al. [41] method. GSH contents were recorded at absorbance 412 nm using 2-nitrobenzoic acid (Sigma-Aldrich, St. Louise, MO, USA) [42]. The H_2_O_2_ decomposition in the presence of CAT was measured spectrophotometrically at 240 nm [43]. SOD activity was recorded at 560 nm using spectrophotometer [44].

### 2.13. Hepatic Glucose-Regulating Enzyme Activities Measurement

The hepatic enzyme sources were prepared following the method of Hulcher and Oleson [45]. The activity of GK enzyme was checked spectrophotometrically at 340 nm according to the method of Davidson and Arion [46]. The changes in G6Pase enzyme activity were recorded spectrophotometrically at 340 nm according to the method of Alegre et al. [47]. The changes in PEPCK activity were recorded spectrophotometrically at 340 nm using the method of Bentle and Lardy [48].

### 2.14. Real-Time RT-PCR Analysis

The AMPKα2, AMPKα1, and ACC1 expressions in the prepared hepatic tissues and PPARα, PPARγ, UCP2, leptin, adiponectin, C/EBPβ, C/EBPα, SREBP1c, and FAS mRNA expressions in the periovarian adipose tissue were measured using real-time RT-PCR based on the previous reports [49,50]. The PCR primer sequences are listed in Table 2. The experimental conditions were as follows: 1 cycle of 94 °C for 10 min and 39 cycles of (94 °C for 15 s, 57 °C for 20 s, and 72 °C for 30 s), and 1 cycle of 72 °C for 10 min. The GAPDH mRNA expression levels were used to normalize the data using comparative threshold cycle method [51].

### 2.15. Histopathology

Histological analyses were performed as demonstrated in our previous reports [11,12,13,15]. Briefly, after organ weights measurements, parts of left kidney, left lateral lobes of liver, splenic lobes of pancreas, abdominal wall-deposited fat pads, and left periovarian fat pads were fixed in 10% neutral buffered formalin. The 3–4 μm serial sections were obtained using microtome (Leica Biosystems, Nussloch, Germany), stained by hematoxylin and eosin (H/E stain), and investigated under a light microscope (Nikon, Tokyo, Japan). Histomorphometry was performed on all pancreatic islets in a unit area (1 mm^2^), and the average value was calculated. In addition, histomorphological measurements were performed in the same way for all experimental groups and compared to each other. Since the size of 10 hepatocytes and adipocytes per replacement tissue was measured by performing histological analysis in all experimental groups, in reality, there were 10 hepatocytes or adipocytes × 10 experimental animals per subject; thus, a total of 100 hepatocytes and adipocytes were measured. Analyses were performed and compared with each group. Therefore, it is judged that the mutual comparison will be reliable to some extent. Alternatively, dehydrated portions of liver in 30% sucrose solutions were stained using oil red. Mean hepatocyte diameters (in H/E staining) were also measured using an automated image analysis on the restricted view fields.

### 2.16. Immunohistochemistry

The prepared serial sectioned pancreatic tissues were immune-stained by an avidin–biotin–peroxidase (ABC) method [11,12,13,15] using guinea pig polyclonal insulin (Abcam, Cambridge, UK; dilution rate: 1:100) or rabbit polyclonal glucagon (Abcam, Cambridge, UK; dilution rate: 1:100) antiserum. The immunohistochemistry analyses were performed as demonstrated in our previous reports [11,12,13,15]. The mean glucagon- and insulin-immunoreactive cells dispersed in the pancreatic parenchyma were counted using an automated image analysis [11,12,13,15], and insulin/glucagon cell ratios were calculated using Equation (4). The histopathologist was blinded to the group distribution during analysis.

Insulin/glucagon cells (ratio) = (mean numbers of insulin-immunoreactive cells/mean numbers of glucagon-immunoreactive cells)(4)

### 2.17. Statistical Analyses

Levene’s test was used for variance homogeneity [52]. In case of nonsignificant deviation observed by the Levene’s test, one-way analysis of variance (ANOVA) followed by Tukey’s honest significant difference (THSD) test was used to determine the significantly different groups. In case of significant deviations observed using Levene’s test, a nonparametric comparison test, Kruskal–Wallis H (KWH) test, was performed. In case of a significant difference noticed in the KWH test, MW test was conducted to determine significantly different groups. Statistical analyses were conducted using SPSS version 23.0 (IBM-SPSS Inc., Chicago, IL, USA). In addition, the percent changes in the test substance-administered mice as compared to HFD control were calculated to better understand the efficacy of test material. The percent changes between NFD and HFD control were also determined to monitor disease inductions by following Equations (5) and (6) [12,13,15].

Percent changes as compared with NFD control (%) = ((Data of HFD control − Data of NFD control)/Data of NFD control) × 100(5)

Percent changes as compared with HFD control (%) = ((Data of test substance administered mice − Data of HFD control)/Data of HFD control) × 100 (6)

## 3. Results

### 3.1. Content of Ellagic Acid in WC Extract

**Figure 1:**
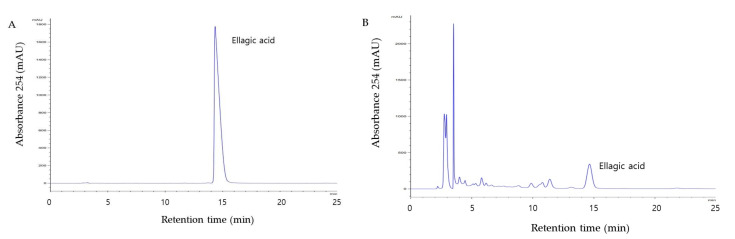
HPLC analysis of chemical standards (**A**) and ellagic acid of WC extract (**B**).

HPLC analysis of WC extract used in this study showed ellagic acid was detected at 4.15 mg/g concentrations (Figure 1).

### 3.2. Effects on Obesity

#### 3.2.1. Change in Weight

**Figure 2:**
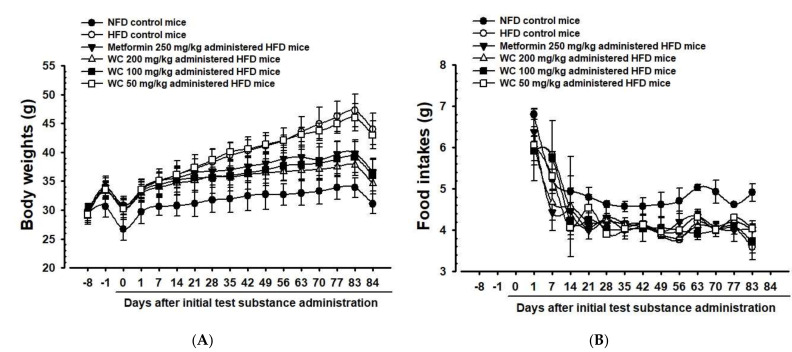
Weekly Body weight (**A**) and food intake (**B**) changes in NFD- or HFD-supplied mice. Values are expressed as Mean ± SD of 10 mice. NFD = normal pellet diet; HFD = 45% Kcal high-fat diet; WC = water chestnut (fruit of *Trapa japonica* Flerov.) extracts. NFD control = vehicle (distilled water) 10 mL/kg orally administered to mice with NFD supply. All animals were fasted overnight before initial test substance administrations and sacrifice. Day 7 means 7 days before initial test material administration. Day 0 means the day of initial test material administration. Day 84 means 24 h after last, 84th, test material administration.

**Figure 3:**
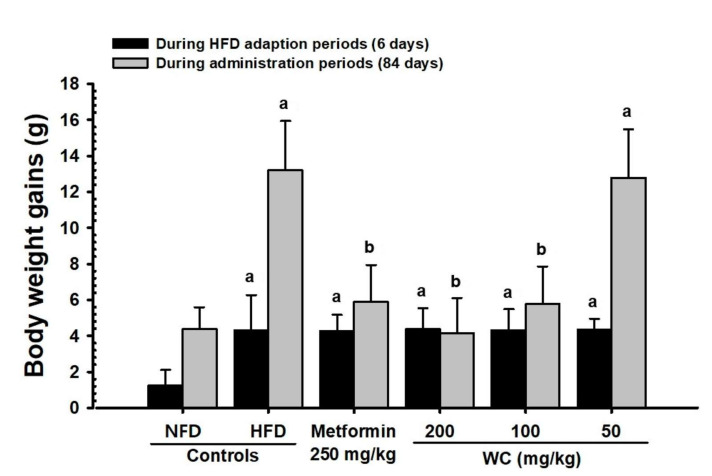
Body weight gains in HFD- and NFD-supplied mice. HFD control mice showed significant increases of body weights as compared with NFD mice from 6 days after HFD supply, and accordingly, the body weight gains during 7 days of HFD adaption and 84 days of administration periods were also significantly increased as compared with NFD-supplied intact control mice, respectively. However, the body weight gains during 84 days of administration were also significantly (*p* < 0.01) and dose-dependently decreased in mice administered 200 and 100 mg/kg WC, and also in metformin-treated (250 mg/kg) mice as compared to those of HFD control, respectively. Values are expressed Means ± SD of 10 mice. HFD = 45% Kcal high-fat diet; NFD = normal pellet diet; WC = water chestnut (fruit of *Trapa japonica* Flerov.) extracts; THSD = Tukey’s honest significant difference, NFD control = vehicle (distilled water) 10 mL/kg orally administered to mice with NFD supply, ^a^ *p* < 0.01 as compared with NFD control by THSD test, ^b^ *p* < 0.01 as compared with HFD control by THSD test.

Only experimental animals showing constant weight gain one week after HFD feeding (average weight of HFD-fed mice: 33.66 ± 1.56 g and NFD-fed mice: 30.65 ± 1.84 g) were selected in the HFD control group. A significant (*p* < 0.01) weight gain compared to the normal control group was observed six days after the HFD supply. Furthermore, weight gains during one-week HFD adaptation period and 84-day test substance administration period also increased significantly (*p* < 0.01) compared to the normal control group. On the other hand, significant (*p* < 0.01 or *p* < 0.05) weight loss was observed 35 days after the start of administration in the group administered 200 mg/kg and 100 mg/kg WC, and 49 days after the start of administration in the metformin (250 mg/kg)-administered group, respectively, compared to the HFD control group. Significant (*p* < 0.01) reduction in weight gain during the 84-day test substance administration was noted in the WC (200 mg/kg and 100 mg/kg)-, and metformin (250 mg/kg)-administered groups, respectively, as compared to the HFD control. In the WC (100 mg/kg)-administered group, body weight and weight gain inhibition effects were noticed as comparable to those of the HFD-supplied control group. However, the WC (50 mg/kg)-administered group showed insignificant changes in body weight and weight gain compared to the HFD control group during the entire experimental period (Table 3; Figure 2A and Figure 3).

#### 3.2.2. Change in Amount of Average Feed Intake

Compared to the normal control group, a significant (*p* < 0.05) reduction in average feed intake was recognized in the HFD control group. However, compared to the HFD control group, a significant change in average feed intake was not recognized in all experimental-substance-administered groups, including HFD-supplied control group (Table 3; Figure 2B).

#### 3.2.3. Changes in Abdominal Fat and Body Fat Mass

**Figure 4:**
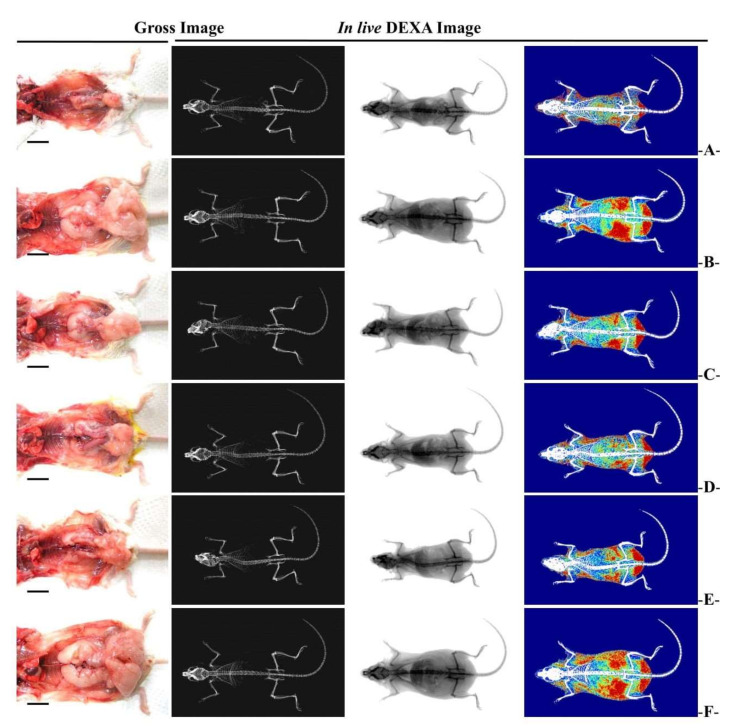
Representative gross body mass and abdominal fat pads with whole-body DEXA images taken from HFD- and NFD-supplied mice. (**A**) = Vehicle (distilled water) 10 mL/kg NFD orally administered to mice (NFD control), (**B**) = vehicle 10 mL/kg HFD orally administered to mice (HFD control), (**C**) = 250 mg/kg of metformin orally administered to mice with HFD supply (Metformin), (**D**) = WC 200 mg/kg orally administered to mice with HFD supply (WC200), (**E**) = WC 100 mg/kg orally administered to mice with HFD supply (WC100), (**F**) = WC 50 mg/kg orally administered to mice with HFD supply (WC50), HFD = 45% Kcal high-fat diet; NFD = normal pellet diet; WC = water chestnut (fruit of *Trapa japonica* Flerov.) extracts; DEXA = dual-energy X-ray absorptiometry. Scale bar = 13.50 mm.

**Figure 5:**
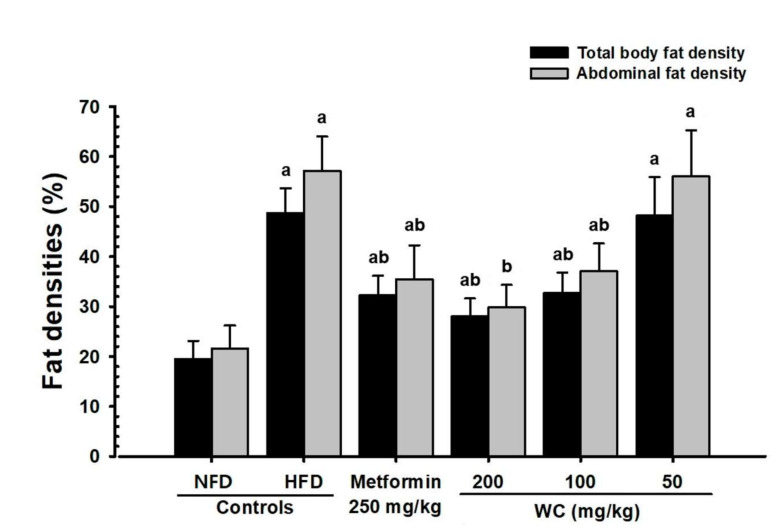
Total body and abdominal fat densities in HFD- and NFD-supplied mice. Values are expressed as means ± SD of 10 mice, HFD = 45% Kcal high-fat diet, NFD = normal pellet diet; WC = water chestnut (fruit of *Trapa japonica* Flerov.) extracts, DEXA = dual-energy X-ray absorptiometry, THSD = Tukey’s honest significant difference, NFD control = vehicle (distilled water) 10 mL/kg NFD orally administered to mice, ^a^ *p* < 0.01 as compared with NFD control by THSD test, ^b^ *p* < 0.01 as compared with HFD control by THSD test.

The significant (*p* < 0.01) increases in abdominal fat and body fat mass were recorded in the HFD control group compared to the normal control group. Whereas, groups administered with WC 200 mg/kg and 100 mg/kg showed significant (*p* < 0.01) reductions in body fat and abdominal accumulated fat mass compared to the HFD control group in a dose-dependent manner. Particularly, WC (100 mg/kg) showed an inhibitory effect on HFD-induced body fat and abdominal fat increase comparable to that of the HFD control group. However, WC (50 mg/kg)-administered group showed insignificant changes in body fat and abdominal fat mass compared to the HFD control group (Figure 4 and Figure 5).

#### 3.2.4. Change in Fat Weight

Significant (*p* < 0.01) increases in the relative and absolute weight of accumulated fat in periovarian and abdominal wall were noticed in the HFD controls compared to the normal control. However, the groups administered WC (200 mg/kg and 100 mg/kg) displayed significant (*p* < 0.01) reductions in accumulated fat weight of periovarian and abdominal wall compared to the HFD control group in a dose-dependent manner. Particularly, WC (100 mg/kg) showed inhibitory effects on the relative and absolute weight gain of HFD-induced accumulation of fat in periovarian and abdominal wall comparable to those of the HFD control group. However, the WC (50 mg/kg)-administered group showed insignificant changes in the relative and absolute weights of accumulated fat in periovarian and abdominal wall compared to the HFD control (Table 4; Figure 4).

#### 3.2.5. Histopathological Changes in Fat Accumulation around the Ovary and in the Abdominal Wall

**Figure 6:**
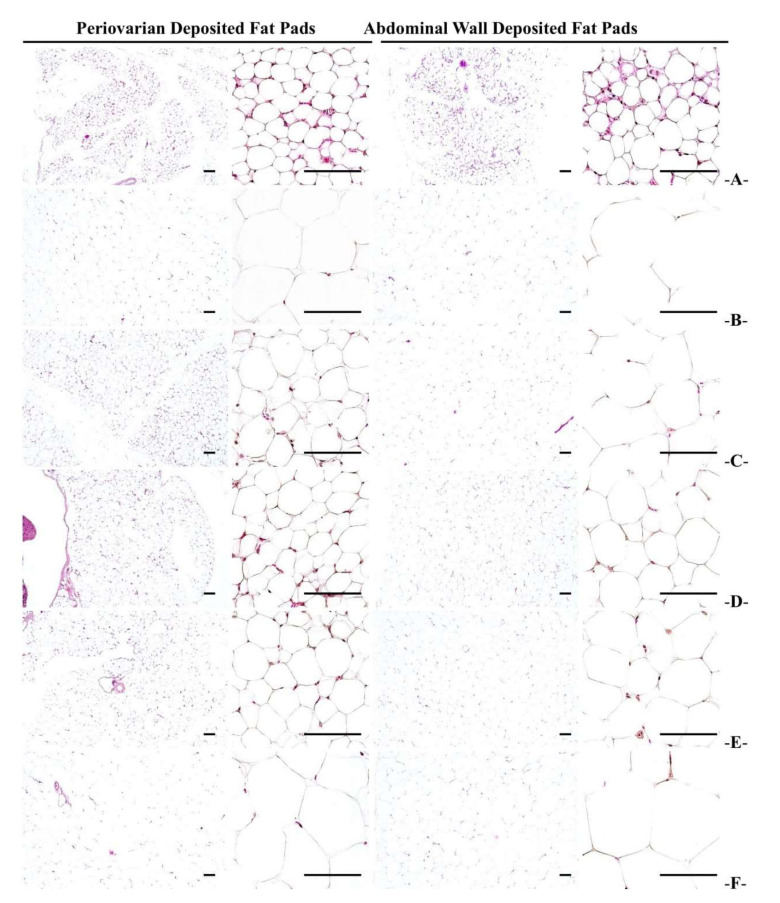
Representative histological images of the adipocytes, taken from NFD- or HFD-supplied abdominal- and periovarian wall-deposited fat pads. (**A**) = Vehicle (distilled water) 10 mL/kg NFD orally administered to mice (NFD control), (**B**) = vehicle 10 mL/kg HFD orally administered to mice (HFD control), (**C**) = 250 mg/kg of metformin orally administered to mice with HFD supply (Metformin), (**D**) = WC 200 mg/kg orally administered mice to with HFD supply (WC200), (**E**) = WC 100 mg/kg orally administered to mice with HFD supply (WC100), (**F**) = WC 50 mg/kg orally administered to mice with HFD supply (WC50), NFD = normal pellet diet; HFD = 45% Kcal high-fat diet; WC = water chestnut (fruit of *Trapa japonica* Flerov.) extracts. All hematoxylin and eosin stain. Scale bars = 80 µm. Low magnification = 40×, and high magnification = 200×.

Remarkable adipocyte hypertrophy was noticed in the HFD control mice, and a significant (*p* < 0.01) increase in the thickness of adipose tissue accumulated in the periovarian and abdominal wall was reported, compared to the normal control. However, the WC (200 mg/kg and 100 mg/kg)-administered mice showed significant (*p* < 0.01) reductions in accumulated adipocyte diameter and fat thickness in the periovarian and abdominal wall in a dose-dependent manner compared to the HFD control group. Particularly, WC (100 mg/kg) indicated inhibitory effects on the increase in the thickness of the adipose tissue accumulated in the periovarian and abdominal wall and the adipocyte diameter in HFD-induced group comparable to those of the HFD control group. However, the WC (50 mg/kg)-administered group showed insignificant changes in the thickness of the adipose tissue accumulated in the abdominal and periovarian wall and the adipocyte diameter compared to the HFD control (Table 5; Figure 6).

#### 3.2.6. Histopathological Changes of Zymogen Granules in the Exocrine Pancreas

**Figure 7:**
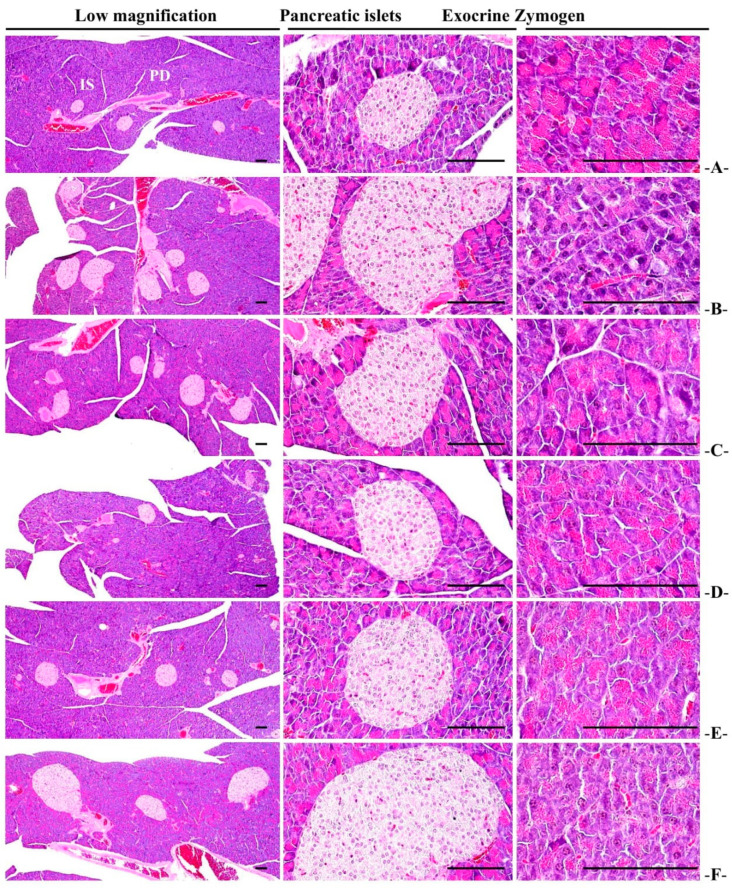
Representative histological images of the pancreas from HFD- or NFD-administered mice. (**A**) = Vehicle (distilled water) 10 mL/kg NFD orally administered to mice (NFD control), (**B**) = vehicle 10 mL/kg HFD orally administered to mice (HFD control), (**C**) = 250 mg/kg of metformin orally administered to mice with HFD supply (Metformin), (**D**) = WC 200 mg/kg orally administered to mice with HFD supply (WC200), (**E**) = WC 100 mg/kg orally administered mice to with HFD supply (WC100), (**F**) = WC 50 mg/kg orally administered to mice with HFD supply (WC50), NFD = normal pellet diet; HFD = 45% Kcal high-fat diet; WC = water chestnut (fruit of *Trapa japonica* Flerov.) extracts, IS = pancreatic islet; PD = pancreatic secretory duct. All hematoxylin and eosin stain, scale bars = 80 µm. Low magnification = 40×, medium magnification = 200×, and high magnification = 400×.

Significant (*p* < 0.01) decreases in the proportion of zymogen granules in the pancreatic exocrine were reported in the HFD control group compared to the normal control. In the WC (200 mg/kg and 100 mg/kg)-administered groups, an increase in the proportion of zymogen granules was significant (*p* < 0.01 or *p* < 0.05) compared to the HFD control in a dose-dependent manner. Particularly, WC (100 mg/kg) showed the inhibitory effect of reducing the proportion of HFD-induced zymogen granules in HFD-induced group comparable to that of the HFD control group. However, the WC (50 mg/kg)-administered group showed insignificant changes in the proportion of zymogen granules in the pancreatic exocrine secretions compared to the HFD control (Table 6; Figure 7).

### 3.3. Antidiabetic Effect

#### 3.3.1. Changes in Blood Sugar

The significant (*p* < 0.01) decreases in blood glucose were reported in the HFD control mice compared to the normal control. In the WC (200 mg/kg and 100 mg/kg)-administered groups, significant (*p* < 0.01) reductions in blood glucose were noted as compared to the HFD control group in a dose-dependent manner. Particularly, WC (100 mg/kg) indicated the inhibitory effect on the HFD-induced blood glucose increase comparable to that of the HFD control group. However, the WC (50 mg/kg)-administered group showed insignificant changes in the blood glucose compared to the HFD control (Table 7).

#### 3.3.2. Changes in Insulin Level in Blood

**Figure 8:**
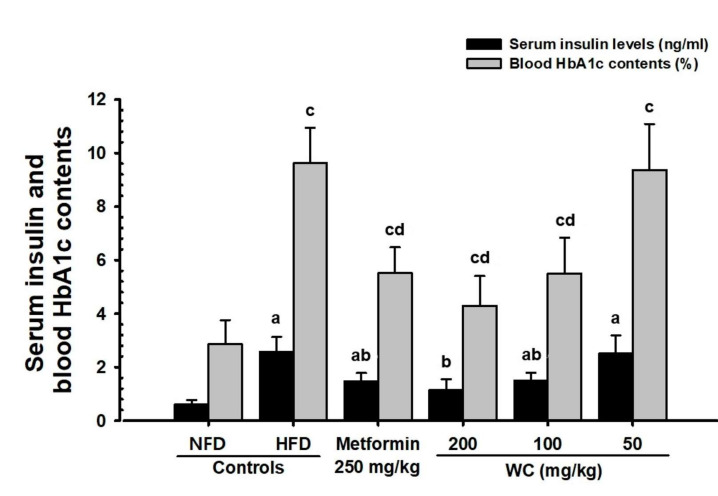
Serum insulin and blood HbA1c contents in HFD- or NFD-administered mice. Values are expressed means ± SD of 10 mice, HFD = 45% Kcal high-fat diet; NFD = normal pellet diet; WC = water chestnut (fruit of *Trapa japonica* Flerov.) extracts; HbA1c = glycated hemoglobin, hemoglobin A1c; THSD = Tukey’s honest significant difference; MW = Mann–Whitney U, NFD control = vehicle (distilled water) 10 mL/kg NFD orally administered to mice, ^a^ *p* < 0.01 as compared with NFD control by THSD test, ^b^ *p* < 0.01 as compared with HFD control by THSD test, ^c^ *p* < 0.01 as compared with NFD control by MW test, ^d^ *p* < 0.01 as compared with HFD control by MW test.

The significant (*p* < 0.01) decreases in blood insulin content were reported in the HFD control mice compared to the normal control. In the WC (200 mg/kg and 100 mg/kg)-administered groups, significant (*p* < 0.01) decreases in blood insulin content were noted as compared to the HFD control in a dose-dependent manner. Particularly, WC (100 mg/kg) indicated the inhibitory effect of HFD-induced increase in blood insulin comparable to that of the HFD control group. However, the WC (50 mg/kg)-administered group showed insignificant changes in the blood insulin content compared to the HFD control (Figure 8).

#### 3.3.3. Changes in the Hba1c Ratio in Blood

The significant (*p* < 0.01) increases in the blood HbA1c ratio were reported in the HFD control mice compared to the normal control. In the WC (200 mg/kg and 100 mg/kg)-administered groups, significant (*p* < 0.01) decreases in HbA1c ratio were observed as compared to the HFD control in a dose-dependent manner. Particularly, WC (100 mg/kg) indicated the inhibitory effect on the increase of the HbA1c ratio in the blood comparable to that of the HFD control group. However, WC (50 mg/kg)-administered group showed insignificant changes in the blood HbA1c ratio compared to the HFD control (Figure 8).

#### 3.3.4. Change in Pancreatic Weight

The significant (*p* < 0.01) reductions in the relative weights of the pancreas were reported in the HFD control mice compared to the normal control. In the WC (200 mg/kg and 100 mg/kg)-administered groups, significant (*p* < 0.01) increases in the relative weight of the pancreas were observed as compared to the HFD control in a dose-dependent manner. Particularly, WC (100 mg/kg) indicated an inhibitory effect on pancreatic relative weight reduction comparable to that of the HFD control group. However, WC (50 mg/kg)-administered group showed insignificant changes in the relative weight of the pancreas compared to the HFD control. In the HFD control and test substance-administered groups, insignificant changes in the absolute weight of the pancreas were observed compared to the normal control (Table 4).

#### 3.3.5. General Histopathological Changes in Pancreatic Islets

Remarkable pancreatic islet proliferation was noticed in HFD control, and significant (*p* < 0.01) increases in the number and average diameters of pancreatic islets were reported in the HFD control group compared to the normal control. In the WC (200 mg/kg and 100 mg/kg)-administered groups, significant (*p* < 0.01) reductions in the number and diameter of pancreatic islets were observed as compared to the HFD control in a dose-dependent manner. Particularly, WC (100 mg/kg) indicated an inhibitory effect on pancreatic relative weight reduction comparable to that of the HFD control. However, the WC (50 mg/kg)-administered group showed insignificant changes in the inhibitory effects on the increases in the number and diameter of HFD-induced pancreatic islets compared to the HFD control group. In the HFD control and test substance-administered groups, insignificant variations in the number and average diameter of pancreatic islets were observed compared to the normal control (Table 6; Figure 7).

#### 3.3.6. Immuno-Histochemical Changes in Pancreatic Islets

**Figure 9:**
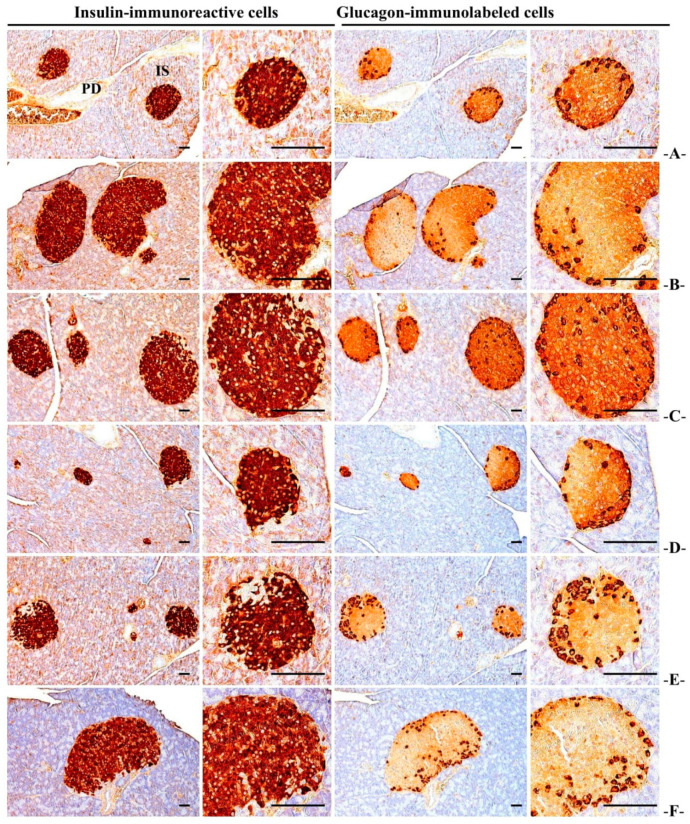
Representative histological images of the insulin- and glucagon-immunoreactive cells in the pancreas from HFD- or NFD-administered mice. (**A**) = Vehicle (distilled water) 10 mL/kg NFD orally administered to mice (NFD control), (**B**) = vehicle 10 mL/kg HFD orally administered to mice (HFD control), (**C**) = 250 mg/kg of metformin orally administered to mice with HFD supply (Metformin), (**D**) = WC 200 mg/kg orally administered to mice with HFD supply (WC200), (**E**) = WC 100 mg/kg orally administered to mice with HFD supply (WC100), (**F**) = WC 50 mg/kg orally administered to mice with HFD supply (WC50), HFD = 45% Kcal high-fat diet; NFD = normal pellet diet; WC = water chestnut (fruit of *Trapa japonica* Flerov.) extracts, IS = pancreatic islet; PD = pancreatic duct. All immunostained by avidin–biotin–peroxidase complex; scale bars = 80 µm. Low magnification = 40×, and high magnification = 200×.

The significant (*p* < 0.01) increases in the number of insulin and glucagon immune response cells and the insulin/glucagon cell ratio were reported in the HFD control mice compared to the normal control. In the WC (200 mg/kg and 100 mg/kg)-administered groups, significant (*p* < 0.01) decreases in the number of insulin and glucagon immune response cells and a decrease in insulin/glucagon cell ratio were observed as compared to the HFD control group in a dose-dependent manner. Particularly, WC (100 mg/kg) indicated the inhibitory effect on the increase in the number of HFD-induced insulin and glucagon immune response cells and the increase in insulin/glucagon cell ratio comparable to that of the HFD control group. However, the WC (50 mg/kg)-administered group show insignificant changes in the inhibitory effects on the increases in the number and diameter of HFD-induced pancreatic islets compared to the HFD control group. In the HFD control and test substance-administered groups, no significant changes in the number of insulin and glucagon immune response cells and the ratio of insulin/glucagon immune response cells were observed compared to the normal control (Table 6; Figure 9).

### 3.4. Effect on Hyperlipidemia

#### 3.4.1. Changes in TG and Total Cholesterol Content in Blood

The significant (*p* < 0.01) increases in total cholesterol and TG content in blood were reported in the HFD control group compared to the normal control group. In the WC (200 mg/kg and 100 mg/kg)-administered groups, significant (*p* < 0.01) reductions in total cholesterol and TG content in the blood were observed as compared to the HFD control group in a dose-dependent manner. Particularly, WC (100 mg/kg) indicated inhibitory effects on the HFD-induced increases in total cholesterol and TG content in the blood comparable to that of the HFD control group. However, the WC (50 mg/kg)-administered group show insignificant variations in the blood total cholesterol and TG content compared to the HFD control (Table 7).

#### 3.4.2. Changes in Blood HDL and LDL Content

Significant (*p* < 0.01) increases in blood LDL content and decrease in blood HDL content were reported in the HFD control group compared to the normal control. In the WC (200 mg/kg and 100 mg/kg)-administered groups, significant (*p* < 0.01) reductions in blood LDL content and increases in blood HDL content were observed as compared to the HFD control group in a dose-dependent manner. Particularly, WC (100 mg/kg) indicated an inhibitory effect on the HFD-induced increase in blood LDL content and HFD-induced decrease in blood HDL content comparable to that of the HFD control group. However, the WC (50 mg/kg)-administered group showed insignificant variations in the blood LDL and HDL content compared to the HFD control (Table 7).

#### 3.4.3. Changes in Lipid Content in Feces

**Figure 10:**
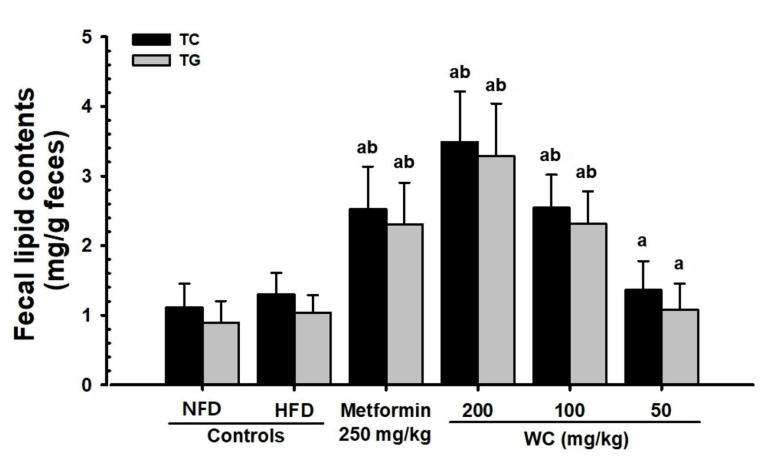
Fecal total cholesterol and TG content in HFD- or NFD-administered mice. Values are expressed means ± SD of 10 mice, HFD = 45% Kcal high-fat diet, NFD = normal pellet diet, WC = water chestnut (fruit of *Trapa japonica* Flerov.) extracts; TG = triglyceride, MW = Mann–Whitney U, NFD control = vehicle (distilled water) 10 mL/kg NFD orally administered to mice, ^a^ *p* < 0.01 as compared with NFD control by MW test, ^b^ *p* < 0.01 as compared with HFD control by MW test.

A slight increase in fecal TG and total cholesterol content was reported in the HFD control mice compared to the normal control. In the WC (200 mg/kg and 100 mg/kg)-administered groups, significant (*p* < 0.01) increases in total cholesterol and TG content in feces were observed as compared to the HFD control in a dose-dependent manner. Particularly, WC (100 mg/kg) indicated an inhibitory effect on the increasing total cholesterol and TG content in feces and the HFD-induced decrease in blood HDL content comparable to that of the HFD control group. However, the WC (50 mg/kg)-administered group showed insignificant variations in TG and total cholesterol content in feces compared to the HFD control (Table 7; Figure 10).

### 3.5. Effects on Liver Damage

#### 3.5.1. Change in Liver Weight

A significant (*p* < 0.01) increase in liver absolute weight was reported in the HFD control group compared to the normal control group. In the WC (200 mg/kg and 100 mg/kg)-administered groups, significant (*p* < 0.01) reductions in liver absolute weights were observed as compared to the HFD control group in a dose-dependent manner. Particularly, WC (100 mg/kg) indicated an inhibitory effect on HFD-induced liver weight increase and HFD-induced decrease in blood HDL content comparable to that of the HFD control. However, the WC (50 mg/kg)-administered group show insignificant variations in absolute liver weight compared to the HFD control. In the HFD control and all test substance-administered groups, insignificant change in liver relative weight was noted compared to the normal control (Table 4).

#### 3.5.2. Changes in ALT, AST, GGT, LDH, and ALP Content in Blood

A significant (*p* < 0.01) increase in blood ALT, AST, GGT, LDH, and ALP content was reported in the HFD control mice compared to the normal control. In the WC (200 mg/kg and 100 mg/kg)-administered groups, a significant (*p* < 0.01) reduction in blood ALT, AST, GGT, LDH, and ALP content was observed as compared to the HFD control group in a dose-dependent manner. Particularly, WC (100 mg/kg) indicated a reducing effect on the increasing HFD-induced blood ALT, AST, GGT, LDH, and ALP content comparable to that of the HFD control group. However, the WC (50 mg/kg)-administered group showed insignificant variations in the blood ALT, AST, GGT, LDH, and ALP contents compared to the HFD control (Table 8).

#### 3.5.3. Histopathological Changes in Liver Fat Change Rate

**Figure 11:**
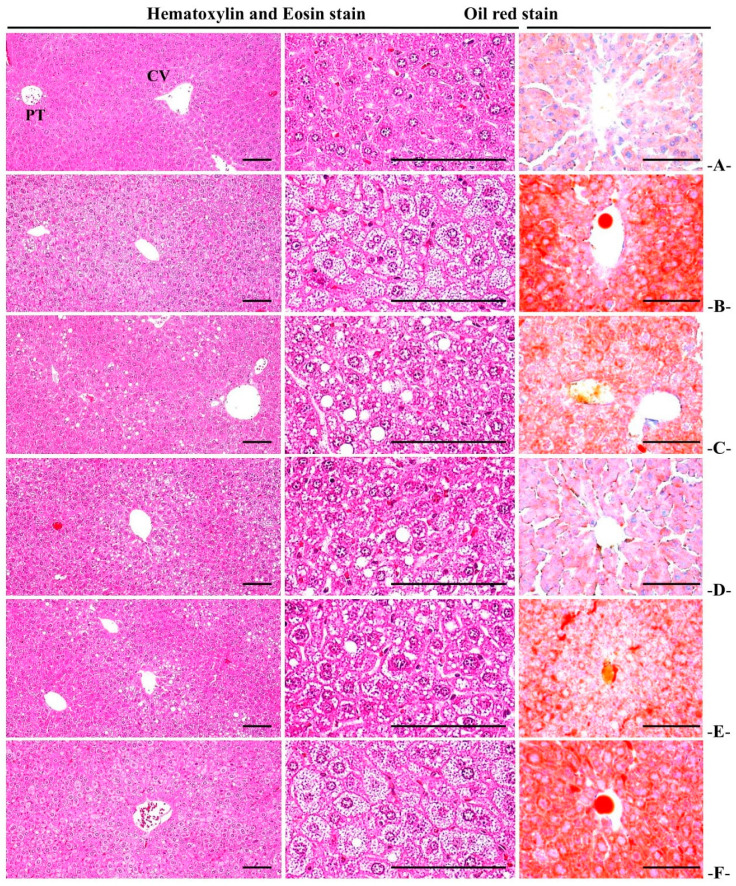
Representative general histological images of the pancreas, taken from HFD- or NFD-administered mice. (**A**) = Vehicle (distilled water) 10 mL/kg NFD orally administered to mice (NFD control), (**B**) = Vehicle 10 mL/kg HFD orally administered to mice (HFD control), (**C**) = 250 mg/kg of metformin orally administered to mice with HFD supply (Metformin), (**D**) = WC 200 mg/kg orally administered to mice with HFD supply (WC200), (**E**) = WC 100 mg/kg orally administered to mice with HFD supply (WC100), (**F**) = WC 50 mg/kg orally administered to mice with HFD supply (WC50), HFD = 45% Kcal high-fat diet; NFD = normal pellet diet; WC = water chestnut (fruit of *Trapa japonica* Flerov.) extracts, CV = central vein; PT = portal triad, scale bars = 80 µm. In HE, low magnification = 100×, high magnification = 400×, and in oil red = 200×.

A fatty liver finding characterized by hepatocellular hypertrophy due to intracellular fat accumulation was reported in the HFD control, and significant (*p* < 0.01) increases in liver fat change rate were reported in the HFD control mice compared to the normal control. In the WC (200 mg/kg and 100 mg/kg)-administered groups, a significant (*p* < 0.01) reduction in liver fat change rate was observed as compared to the HFD control group in a dose-dependent manner. Particularly, WC (100 mg/kg) indicated an inhibitory effect on the increasing HFD-induced liver fat change rate comparable to that of the HFD control. However, the WC (50 mg/kg)-administered group showed insignificant variations in the liver fat change rate compared to the HFD control (Table 9; Figure 11).

#### 3.5.4. Histopathological Changes in Liver Cell Diameter

Significant (*p* < 0.01) increases in hepatocyte diameters were reported in the HFD control group by the fatty liver findings characterized by hepatocellular hypertrophy due to remarkable intracellular fat accumulation. In the WC (200 mg/kg and 100 mg/kg)-administered mice, significant (*p* < 0.01) decreases in mean hepatocyte diameters were noted as compared to the HFD control group in a dose-dependent manner. Particularly, WC (100 mg/kg) indicated an inhibitory effect on the HFD-induced mean hepatocyte diameter increase comparable to that of the HFD control group. However, the WC (50 mg/kg)-administered group showed insignificant variations in the mean hepatocyte diameter compared to the HFD control (Table 9; Figure 11).

### 3.6. Effects on Kidney Damage

#### 3.6.1. Change in Height

Significant (*p* < 0.01) increases in absolute kidney weights were reported in the HFD control group by the fatty liver findings characterized by hepatocellular hypertrophy due to remarkable intracellular fat accumulation. In the WC (200 mg/kg and 100 mg/kg)-administered groups, significant (*p* < 0.01) reductions in the absolute kidney weights were observed as compared to the HFD control group in a dose-dependent manner. Particularly, WC (100 mg/kg) showed an inhibitory effect on HFD-induced kidney absolute weight increase comparable to that of the HFD control. However, the WC (50 mg/kg)-administered group showed insignificant variations in the mean hepatocyte diameter compared to the NFD and HFD control groups (Table 9; Figure 11). In the HFD-supplied group and all test-administered groups, no significant change in kidney relative weight was observed compared to the normal control (Table 4).

#### 3.6.2. Changes in Blood Creatinine and BUN Content

A significant (*p* < 0.01) increase in blood BUN and creatinine content was reported in the HFD control group by the fatty liver findings characterized by hepatocellular hypertrophy due to remarkable intracellular fat accumulation. In the WC (200 mg/kg and 100 mg/kg)-administered groups, significant (*p* < 0.01) reductions in blood BUN and creatinine content were observed as compared to the HFD control group in a dose-dependent manner. Particularly, WC (100 mg/kg) indicated an inhibitory effect on HFD-induced blood BUN and creatinine content increase comparable to that of the HFD control group. However, the WC (50 mg/kg)-administered group showed insignificant variations in blood creatinine and BUN content compared to the HFD control (Table 8).

#### 3.6.3. Renal Histopathological Changes

**Figure 12:**
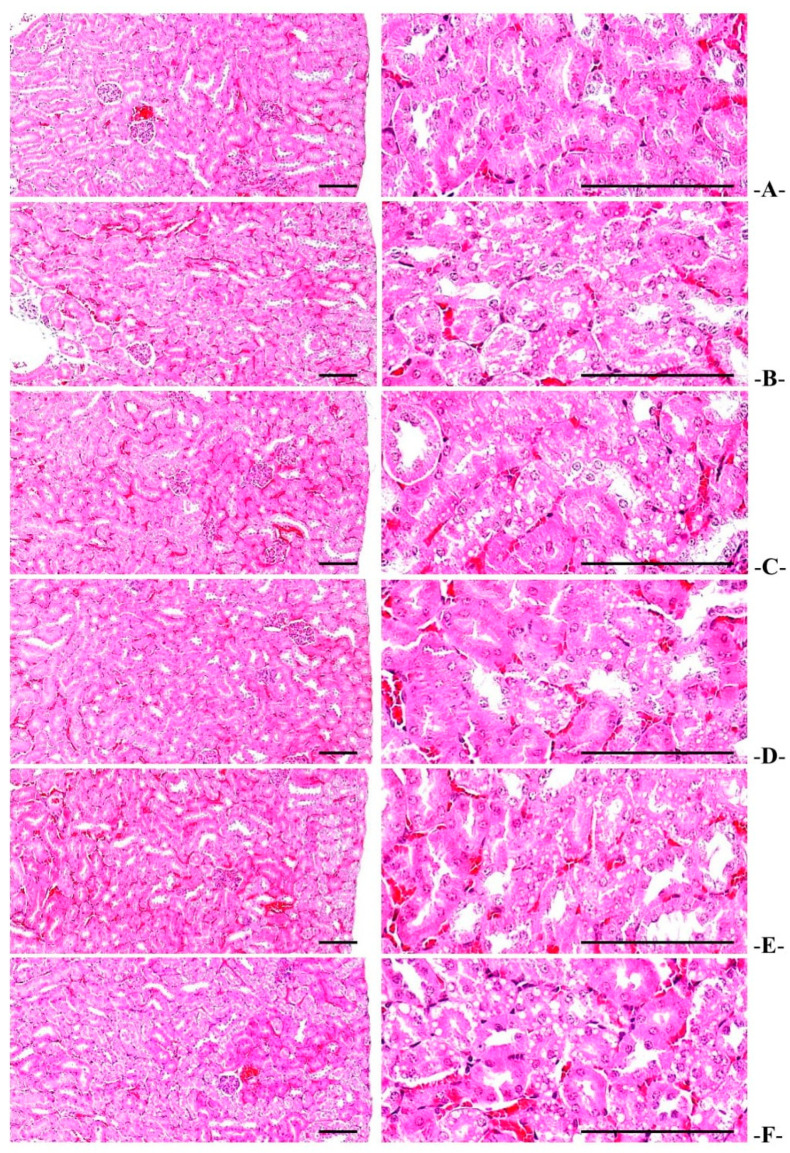
Representative histological images of the kidney from HFD- or NFD-administered mice. (**A**) = Vehicle (distilled water) 10 mL/kg NFD orally administered to mice (NFD control), (**B**) = vehicle 10 mL/kg HFD orally administered to mice (HFD control), (**C**) = 250 mg/kg of metformin orally administered to mice with HFD supply (Metformin), (**D**) = WC 200 mg/kg orally administered to mice with HFD supply (WC200), (**E**) = WC 100 mg/kg orally administered to mice with HFD supply (WC100), (**F**) = WC 50 mg/kg orally administered to mice with HFD supply (WC50), NFD = normal pellet diet, HFD = 45% Kcal high-fat diet, WC = water chestnut (fruit of *Trapa japonica* Flerov.) extracts, All hematoxylin and eosin stain, scale bars = 80 µm. Low magnification = 100×, and high magnification = 400×.

Renal degeneration characterized by vacuolization of the tubules due to accumulation of fat droplets was reported in the HFD control group, and significant (*p* < 0.01) increases in the number of denatured tubules were reported in the HFD control mice compared to the normal control. In the WC (200 mg/kg and 100 mg/kg)-administered groups, a significant (*p* < 0.01 or *p* < 0.05) reduction in the number of denatured tubules was observed as compared to the HFD control group in a dose-dependent manner. Particularly, WC (100 mg/kg) displayed an inhibitory effect on the increases in the number of HFD-induced denatured tubules comparable to that of the HFD control group. However, the WC (50 mg/kg)-administered group showed insignificant variations in the number of denatured tubules compared to the HFD control (Table 9; Figure 12).

### 3.7. Effect on the Liver Antioxidant Defense System

#### 3.7.1. Changes in Lipid Peroxidation

Significant (*p* < 0.01) increases in liver lipid peroxidation, that is, an increase in MDA content, were reported in the HFD control mice by the fatty liver findings characterized by hepatocellular hypertrophy due to remarkable intracellular fat accumulation. In the WC (200 mg/kg and 100 mg/kg)-administered mice, significant (*p* < 0.01) reductions in liver lipid peroxidation levels were observed as compared to the HFD control in a dose-dependent manner. Particularly, WC (100 mg/kg) displayed an inhibitory effect on HFD-induced liver lipid peroxidation increase comparable to that of the HFD control. However, the WC (50 mg/kg)-administered group showed insignificant variations in the MDA content in liver tissue compared to the HFD control (Table 10).

#### 3.7.2. Changes in GSH Content in Liver Tissue

Significant (*p* < 0.01) reductions in the content of GSH were reported in the HFD control group. In the WC (200 mg/kg and 100 mg/kg)-administered mice, significant (*p* < 0.01) increases in the GSH content in liver tissue were noticed as compared to the HFD control group in a dose-dependent manner. Particularly, WC (100 mg/kg) displayed an inhibitory effect on the reducing HFD-induced liver GSH content comparable to that of the HFD control. However, the WC (50 mg/kg)-administered group showed insignificant changes in GSH content in liver tissue compared to the HFD control (Table 10).

#### 3.7.3. Changes in CAT and SOD Activity in Liver Tissue

Significant (*p* < 0.01) reductions in the endogenous antioxidant enzymes, SOD and CAT activity in liver tissue, were observed in the HFD control mice. In the WC (200 mg/kg and 100 mg/kg)-administered mice, a significant (*p* < 0.01) increase in CAT and SOD activity in liver tissues was observed as compared to the HFD control group in a dose-dependent manner. Particularly, WC (100 mg/kg) displayed an inhibitory effect on the reduction of HFD-induced liver CAT and SOD activity comparable to that of the HFD control. However, the WC (50 mg/kg)-administered group showed insignificant variations in SOD and CAT activities in the liver tissue compared to the HFD control (Table 10).

### 3.8. Changes in Liver Enzyme Activity Related to Sugar Metabolism

#### 3.8.1. Changes in GK Activity in Liver Tissue

A significant (*p* < 0.01) reduction in GK enzyme activity in liver tissue, which is a glycolytic enzyme, was reported in the HFD control mice. In the WC (200 mg/kg and 100 mg/kg)-administered groups, a significant (*p* < 0.01) increase in GK activity in liver tissue was observed as compared to the HFD control group in a dose-dependent manner. Particularly, WC (100 mg/kg) displayed an inhibitory effect on the HFD-induced reduction in liver GK activity comparable to that of the HFD control. However, the WC (50 mg/kg)-administered group showed insignificant variations in the GK activity in liver tissue compared to the HFD control (Table 11).

#### 3.8.2. Changes in G6Pase Activity in Liver Tissue

Significant (*p* < 0.01) increases in G6Pase activity in liver tissue were reported in the HFD control group. In the WC (200 mg/kg and 100 mg/kg)-administered groups, a significant (*p* < 0.01) decrease in G6Pase activity in liver tissue was observed as compared to the HFD control group in a dose-dependent manner. Particularly, WC (100 mg/kg) indicated an inhibitory effect on the HFD-induced increase in G6Pase activity in liver tissue comparable to that of the HFD control. However, the WC (50 mg/kg)-administered group showed insignificant variations in the G6Pase activity in liver tissue compared to the HFD control (Table 11).

#### 3.8.3. Changes in PEPCK Activity in Liver Tissue

Significant (*p* < 0.01) increases in liver PEPCK activity were reported in the HFD control mice. In the WC (200 mg/kg and 100 mg/kg)-administered mice, a significant (*p* < 0.01) decrease in PEPCK activity in liver tissues was observed as compared to the HFD control group in a dose-dependent manner. Particularly, WC (100 mg/kg) displayed an inhibitory effect on the HFD-induced increase in liver PEPCK activity comparable to that of the HFD control. However, the WC (50 mg/kg)-administered group showed insignificant variations in PEPCK activity in liver tissue compared to the HFD control (Table 11).

### 3.9. Changes in Gene Expression Related to Lipid Metabolism

#### Changes in mRNA Expression in Liver Tissue

A significant (*p* < 0.01) increase in ACC1 mRNA expression in liver tissue leptin, mRNA expression of C/EBPβ, C/EBPα, SREBP1c, FAS, and PPARγ in adipose tissue, and decrease in UCP2, AMPKα2, adiponectin, AMPKα1, and PPARα mRNA expressions were reported in the HFD control mice. In the WC (200 mg/kg and 100 mg/kg)-administered groups, significant (*p* < 0.01 or *p* < 0.05) decreases in the expression of ACC1 mRNA in liver tissue and of and mRNA expression of leptin, C/EBPβ, C/EBPα, SREBP1c, FAS, and PPARγ, and increased mRNA expressions of UCP2, AMPKα2, adiponectin, AMPKα1, adiponectin, and PPARα in adipose tissue were observed as compared to the HFD control group in a dose-dependent manner. Particularly, WC (100 mg/kg) displayed inhibitory properties on the HFD-induced increase in ACC1 mRNA expression in liver tissue and on the decrease in AMPKα1 and AMPKα2 mRNA expression comparable to those of the HFD control group (Table 12). Furthermore, WC (100 mg/kg) displayed the HFD-induced adipose tissue increase in leptin, C/EBPβ, C/EBPα, SREBP1c, FAS, and PPARγ mRNA expression and decrease in PPARα, adiponectin, and UCP2 mRNA expressions comparable to those of HFD control group (Table 13). However, the WC (50 mg/kg)-administered group showed insignificant variations in the expression of AMPKα2, AMPKα1, and ACC1 mRNA in liver tissues and of leptin, C/EBPβ, C/EBPα, adiponectin, SREBP1c, FAS, UCP2, PPARα, and PPARγ in the adipose tissue compared to the HFD control group.

## 4. Discussion

With increasing prevalence of metabolic syndrome worldwide, the occurrence of NAFLD is rapidly increasing [53]. Fat accumulation in hepatic cells during NAFLD is known to cause liver cell damage, inflammation, and fibrosis, which leads to more severe NASH, liver cirrhosis, and hepatocellular carcinoma [54,55]. There are currently no definitive drugs for the treatment of metabolic syndrome [54] except some drugs used to control metabolic syndrome [56]. Furthermore, the use of the currently available drugs is limited due to various associated side effects [16]. Lifestyle control, such as weight loss through exercise therapy, is mainly used for the treatment of metabolic syndrome [54]. Thus, there is an urgent need to develop a drug that has low side effects even when used for a long time and that can suppress the overall metabolic syndrome. In this context, WC has shown proven antioxidant and anti-inflammatory effects for improving complications related to diabetes and obesity [24,25,26]. It has higher potential to be developed as a new drug material or functional food for metabolic syndrome including obesity. Therefore, in the present study, we investigated the anti-type II diabetic and anti-obesity effects of WC extracts using a 45% Kcal HFD mouse model as part of the development of natural products-derived functional foods or new natural drugs to improve related complications, including diabetes and obesity.

Diabetic complications including obesity, diabetes, and NAFLD were markedly suppressed in a dose-dependent manner compared to the HFD control mice by continuous oral administration of WC (200 mg/kg and 100 mg/kg) over a period of 84 days. Furthermore, the suppression of oxidative stress through lipid peroxidation of the liver, the activity of the antioxidant defense system, the normalization of the enzyme activity related to sugar metabolism in the liver tissue, and the normalization of the expression of genes related to fat metabolism were also significant in the WC (200 mg/kg and 100 mg/kg)-administered groups in a dose-dependent manner as compared to the HFD control group. Particularly, the WC (100 mg/kg)-administered group showed inhibitory effects on HFD-induced diabetes and related obesity, hyperlipidemia, diabetic nephropathy, and NAFLD, compared to the HFD control. Therefore, at least under the conditions of this experiment, WC (100 mg/kg) is comparable to metformin at a dose of 250 mg/kg.

In rodents such as mice, obesity is caused by HFD supply, which is directly linked to type II diabetes identified by high blood sugar, insulin resistance, fatty liver, renal degeneration, and hyperlipidemia [12,13,15]. Therefore, the current rodent model by HFD supply is considered as one of the most widely used animal models for the advancement of treatments for diabetes, obesity, and related metabolic syndrome [12,13,14]. In the HFD control group, a significant increase in body weight compared to the normal control was observed six days after feeding the HFD. The WC (100 mg/kg)-administered group showed a HFD-supplied body weight and weight gain inhibition effect comparable to that of the HFD control. This shows that WC (100 mg/kg) has an excellent inhibitory effect on HFD-induced weight gain, comparable to that of metformin at a dose of 250 mg/kg.

Fat accumulation is the most common phenomenon that causes obesity, and during histopathological examination, fat cell enlargement is caused [12,13]. Changes in endocrine and adipokine secretion in the adipose tissue lead to complex diseases such as insulin resistance and obesity [12,13]. During this experiment, the WC (100 mg/kg)-administered group showed inhibitory effects on HFD-induced fat accumulation and adipocyte hypertrophy comparable to those of HFD control. This shows dose-dependent symptoms-relieving effects of WC (100 mg/kg) on obesity comparable to those of HFD control in HFD-supplied mice. Similar to previous studies [12,13], the HFD control mice showed significant decreases in feed intake compared to the normal control. However, in terms of caloric value, since the caloric value (4.73 kcal/g) of HFD used in this experiment was relatively higher than that of NFD (4.00 kcal/g), it was evaluated that it did not have a significant effect on the induction of obesity. In addition, no significant changes in feed intake were recognized in all test-substance-supplied mice, including metformin 250 mg/kg, compared to the HFD control group; thus, it shows that the pharmacological effects recognized in this experiment were not due to simple feed intake reduction.

The zymogen granules of the exocrine part of the pancreas contain enzymes mainly involved in the digestion of fats and proteins [57]. When obesity occurs, in the pancreas, it is known to cause a significant reduction of these zymogen granules along with fat accumulation [58]. The histopathological analysis showed that the proportions of zymogen granules in the pancreas were significantly suppressed in a dose-dependent manner in the WC (200 mg/kg and 100 mg/kg)-administered mice, particularly in the WC (100 mg/kg)-administered group. This shows that the WC (100 mg/kg) inhibited fat absorption by mediating the regulation of pancreatic fat digestive enzyme secretion in HFD mice in a dose-dependent manner comparable to metformin at as dose of 250 mg/kg. This proves that WC has obesity-improving properties.

HbA1c is produced by long-term hyperglycemia-exposed red blood cells and is clinically used as an important index to determine long-term hyperglycemia [59,60]. During type II diabetes progression, an increase in HbA1c content associated with long-term hyperglycemia is generally observed as part of insulin resistance, along with an increase in the insulin content in the blood [12,13]. In the HFD control group of this experiment, a remarkable increase in blood sugar, blood insulin, and HbA1c content, as well as an increase in the number and dilation of pancreatic islets, and an increase in insulin, glucagon, and insulin/glucagon cells were recognized histopathologically, and typical insulin resistance type II diabetes was triggered. However, the WC (200 mg/kg and 100 mg/kg)-administered mice, particularly the WC (100 mg/kg)-administered mice, showed significant blood glucose reducing effects through pancreatic endocrine control in HFD-supplied mice compared to the metformin at a dose of 250 mg/kg.

When hyperglycemia persists, hyperlipidemia results in the form of complications such as HDL decreases along with an increase in LDL, total cholesterol and TG content become more problematic [17]. Therefore, the anti-hyperlipidemic effect of candidate substances has been focused on increasing HDL content along with decreasing blood LDL, total cholesterol, and TG content [12,13]. During this experiment, the WC (200 mg/kg and 100 mg/kg)-administered mice, particularly (100 mg/kg)-administered mice, showed a significant decrease in blood TG, LDL, and total cholesterol content and an increase in HDL content compared to the HFD control. This clearly shows that WC (100 mg/kg) inhibits HFD-induced hyperlipidemia in a comparable fashion to HFD control. Similarly, the metformin (250 mg/kg) administration showed an increase in fecal TG and total cholesterol content and histopathological increase in the zymogen content in the exocrine part of the pancreas; thus, the hyperlipidemia improvement effect of WC was once again determined by the increase in lipid secretion due to the inhibition of digestion and absorption of lipids through the regulation of the secretion of digestive enzymes in the pancreas.

During diabetes progression, the symptoms of diabetic liver disease such as the content of AST, ALT, ALP, LDH, and GGT in blood increase due to fat accumulation and degeneration of hepatocytes along with an increase in liver weight due to damage to hepatocytes caused by fibrosis and abnormal glycolysis [12,13]. These comprise the most common diagnostic index in the blood chemistry to measure liver damage [61]. During this experiment, the WC (200 mg/kg and 100 mg/kg)-administered mice, particularly 100 mg/kg administered mice, showed significant inhibition of the increase in the absolute weight of HFD-induced liver and blood AST, ALT, ALP, LDH, and GGT contents in a dose-dependent manner. This indicates a dose-dependent HFD-induced NAFLD improvement effect of WC (100 mg/kg) in the mouse model as comparable to metformin at a dose of 250 mg/kg.

Diabetic nephropathy is caused by glomerular atrophy and fibrosis, inflammatory cell infiltration, and tubular necrosis, and results in increasing blood BUN and creatinine content [12,13]. Among those, the content of BUN and creatinine in the blood is the most representative blood chemical indicator of the state of kidneys [61]. In the HFD control group, an increase in blood BUN and creatinine content was noticed with a remarkable increase in the absolute kidney weight, and a tubular vacuolization characteristic characterized by infiltration of fat droplets was observed histopathologically. However, the diabetic nephropathy was significantly suppressed in a dose-dependent manner in the WC (200 mg/kg and 100 mg/kg)-administered mice, particularly in (100 mg/kg)-administered mice, comparable to the metformin at a dose of 250 mg/kg.

The inhibition of antioxidant defense mechanisms by free radicals is important in inducing diabetes and related complications [62,63]. Various toxic substances formed by lipid peroxidation, particularly reactive oxygen species (ROS), cause destruction of surrounding tissues [64]. GSH is a representative endogenous antioxidant, and it is known to inhibit various tissue damages by reducing the amount of free radicals formed in cells by acting as a representative antioxidant indictor in tissue [65]. SOD is a representative antioxidant enzyme in the cell and plays an important role in removing various oxidizing substances from cells, and CAT is also an enzyme that converts H_2_O_2_, a strong active oxidizing substance, into H_2_O [66]. Therefore, lipid peroxidation, reduction of GSH content, and inhibition of SOD and catalase activity are very important in the treatment of diabetes and related disorders [67]. Similar to previous studies [68,69], the increase in MDA content in the liver parenchyma due to lipid peroxidation and a decrease in the endogenous antioxidant GSH content and SOD/CAT activities were observed in the HFD control group. Conversely, this disturbance of the antioxidant defense system and related lipid peroxidation findings were markedly suppressed in a dose-dependent manner in the WC (200 mg/kg and 100 mg/kg)-administered mice, particularly in the 100 mg/kg administered mice, comparable to that of metformin at a dose of 250 mg/kg.

GK, being a representative liver tissue enzyme responsible for blood sugar control [70], promotes the utilization of blood sugar as energy or induces storage as glycogen in liver tissue, thereby reducing blood sugar [71]. Contrarily, G6Pase and PEPCK are enzymes in the liver tissue involved in gluconeogenesis and induce the release of sugar in the liver tissue, thereby increasing blood sugar [72,73]. In general, as a hyperglycemic state results, an increase in G6Pase and PEPCK activity is accompanied with a significant decrease in GK activity in HFD-fed mice [13,68]. During this experiment, significant decreases in GK activity in liver tissue and increases in PEPCK and G6Pase activity were noticed in the HFD control mice compared to the normal control. However, the changes in the GK, G6Pase, and PEPCK activity were markedly inhibited in a dose-dependent manner in the WC (200 mg/kg and 100 mg/kg)-administered mice, particularly in the WC (100 mg/kg)-administered mice, comparable to that of metformin at a dose of 250 mg/kg.

To understand the mechanism of action of candidates for diabetes and related complications including NAFLD, mRNA expressions for genes involved in fat metabolism in adipose tissue and liver were studied. Increased expression and activity of AMPK in liver and adipose tissue is known as one of the most important cell signaling systems, promoting fat oxidation, and inhibiting liposynthesis and glucose production, thereby regulating blood sugar and fat metabolism [74,75]. Therefore, it is of utmost importance to observe the mRNA expression patterns for the genes of the AMPK and proteins involved in the AMPK signal transduction system in the liver and adipose tissue. Increased expression of AMPK is always consistent with an increase in activity of the body’s endogenous antioxidant defense system [20] and an effective modulating effect of glucose metabolism-related activity [18,19]. Similar to Sung et al. [50], the HFD control group showed decreased mRNA expression of AMPKα2 and AMPKα1 in liver and an increase in ACC1 mRNA expression in liver, an increase in mRNA expressions of C/EBPβ, C/EBPa, SREBP1c, FAS, PPARγ, and leptin in adipose tissue, and a decrease in PPARα, adiponectin, and UCP2 mRNA expression in adipose tissue. However, the WC (200 mg/kg and 100 mg/kg)-administered mice, particularly (100 mg/kg)-administered mice, significantly inhibited the expression changes of AMPK and lipid metabolism-related gene mRNA in HFD-induced adipose and liver tissue. These results clearly indicate that WC (100 mg/kg) exhibits dose-dependent effects on lipid metabolism through inhibition of lipid synthesis and increase of fatty acid oxidation in a dose-dependent manner in the HFD-fed mice, comparable to metformin at a dose of 250 mg/kg.

The fruit shell of water chestnut (*Trapa japonica*) has been reported effective in controlling postprandial hyperglycemia and exerts an antioxidant effect in streptozotocin (STZ)-induced diabetic rats [31]. Kang and coworkers focused on diabetes only in the STZ-induced model, and in the current study we used the high-fat diet diabetes model, which shows the improved effects on metabolic syndrome including obesity and NAFLD. During the current study, ellagic acid was a functional component of water chestnut extracts. Ellagic acid has previously shown antidiabetic activity in several studies [76,77,78]. Malini and coworkers showed potent anti-hyperglycemic effects of ellagic acid in STZ-induced diabetic female albino Wistar rats, normalizing glucose levels and carbohydrate metabolic enzymes in the rats [76]. In another study, ellagic acid exhibited renal protective effects in high-fat diet/low-dose streptozotocin (HFD/STZ)-induced type 2 diabetic Wistar albino male rats partly through anti-hyperglycemic response following attenuation of inflammatory processes via inhibition of NF-jB pathway [77]. In a recent study by Farhood and colleagues, ellagic acid treatment exhibited decreased blood glucose levels, modulation of inflammation response, improved neurotrophic support and ameliorated the neuronal loss in streptozotocin (STZ)-induced male Wistar diabetic rats [78].

The limitations of this study are as follows: due to the fact that this study was conducted in rodents, the response in humans could be different. Therefore, additional research is needed in clinical trials. In this experiment, the estrus cycle of the animal was not considered, and there may be a difference in body weight depending on the estrus cycle. In addition, there is a lack of a clear mechanism for alleviating obesity and its complications. Thus, it is judged that further research is necessary to check the bioactivity of the indicated substance.

## 5. Conclusions

As part of the development of natural product-derived functional foods or natural product new drugs to improve HFD-induced obesity, diabetes-related disorders, and NAFLD, WC (200, 100, and 50 mg/kg) dose-dependently improved diabetes and related complications such as hyperlipidemia, NAFLD, and diabetic kidney. The symptom improvement effect was evaluated by comparing with metformin (250 mg/kg) using 45% Kcal HFD-supplied mice, which are most used as experimental animal models for mild Type II diabetes. HFD-induced obesity, diabetic-related complications, and NAFLD were significantly suppressed in a dose-dependent manner by continuous oral administration of WC (200 mg/kg and 100 mg/kg) for a period of 84 days. Inhibition of oxidative stress, activity of antioxidant defense system, normalization of enzyme activity related to glucose metabolism in liver tissue, and normalization of gene expression related to fat metabolism were also reported in a dose-dependent manner in the WC (200 mg/kg and 100 mg/kg)-administered groups. Particularly, the WC (100 mg/kg)-administered group showed inhibitory effects on HFD-induced obesity and diabetes-related disorders, hyperlipidemia, NAFLD, and diabetic nephropathy comparable to those of the metformin (250 mg/kg)-administered group. However, the WC (50 mg/kg)-administered group showed no significant effect on improving diabetic complications, diabetes, and NAFLD compared to the HFD control group. Therefore, at least under the conditions of this experiment, WC at a dose of 100 mg/kg is comparable to metformin (250 mg/kg). Furthermore, it showed hyperlipidemia-, NAFLD-, and kidney damage-improving effects in a dose-dependent manner. This study suggests that the WC at an appropriate dose has higher potential to be developed as an effective therapeutic drug or functional food for diabetes and various diabetic complications including NAFLD.

## Figures and Tables

**Table 1:** Formulations of high-fat and normal pellet diets used in this study.

Compositions	High-Fat Diets	Normal Pellet Diets
Ingredient (g/kg)		
Sucrose	172.8	500
Casein	200	200
Lard	177.5	0
Corn starch	72.8	150
Cellulose	50	50
Mineral mixture	35	35
Soybean Oil	25	50
Vitamin mixture	10	10
Choline bitartrate	2	2
L-Cystein	3	3
Fat (% kcal)	45	16
Carbohydrate (% kcal)	35	64
Protein (% kcal)	20	20
Energy (kcal/g)	4.73	4.00

**Table 2:** Oligonucleotides for real-time RT-PCR used in this study.

Target	5′–3′	Sequence	GenBank Accession Number
PPARα	Forward Reverse	ATGCCAGTACTGCCGTTTTC GGCCTTGACCTTGTTCATGT	NM_011144
PPARγ	Forward Reverse	AGTGGAGACCGCCCAGG GCAGCAGGTTGTCTTGGATGT	NM_001127330
Leptin	Sense Antisense	CCAAAACCCTCATCAAGACC GTCCAACTGTTGAAGAATGTCCC	NM_008493
UCP2	Sense Antisense	CCGCATTGGCCTCTACGACTCT CCCCGAAGGCAGAAGTGAAGTG	NM_011671
Adiponectin	Sense Antisense	CCCAAGGGAACTTGTGCAGGTTGGATG GTTGGTATCATGGTAGAGAAGAAAGCC	NM_009605
C/EBPα	Sense Antisense	TGGACAAGAACAGCAACGAGTAC CGGTCATTGTCACTGGTCAACT	NM_001287523
C/EBPβ	Sense Antisense	AAGCTGAGCGACGAGTACAAGA GTCAGCTCCAGCACCTTGTG	NM_001287739
SREBP1c	Sense Antisense	AGCCTGGCCATCTGTGAGAA CAGACTGGTACGGGCCACAA	XM_006532714
FAS	Sense Antisense	GCTGCGGAAACTTCAGGAAAT AGAGACGTGTCACTCCTGGACTT	NM_007988
ACC1	Sense Antisense	GCCATTGGTATTGGGGCTTAC CCCGACCAAGGACTTTGTTG	NM_133360
AMPKα1	Sense Antisense	AAGCCGACCCAATGACATCA CTTCCTTCGTACACGCAAAT	XM_011245321
AMPKα2	Sense Antisense	GATGATGAGGTGGTGGA GCCGAGGACAAAGTGC	NM_178143
GAPDH	Sense Antisense	CATCTTCCAGGAGCGAGACC TCCACCACCCTGTTGCTGTA	NM_008084

RT-PCR = reverse transcription polymerase chain reaction; PPAR = peroxisome proliferator-activated receptor; UCP = mitochondrial uncoupling protein; C/EBP = CCAAT-enhancer-binding protein; SREBP = sterol regulatory element-binding protein; ACC1 = acetyl-CoA carboxylase 1; FAS = fatty acid synthase; AMPK = 5′ adenosine monophosphate-activated protein kinase; GAPDH = glyceraldehydes 3-phosphate dehydrogenase.

**Table 3:** Changes of body weights and mean daily food consumption patterns in HFD- and NFD-supplied mice.

Times Groups	Body Weights (g) at Days after Initial Test Substance Treatment				Body Weight Gains during		Mean Daily Food Consumption (g)
	8 Days before (A)	1 Day before (B)	0 Day * (C)	84 Days * (D)	Adapt Period (B−A)	Administration Period (D−C)	
Controls							
NFD	29.41 ± 1.52	30.65 ± 1.84	26.72 ± 2.00	31.09 ± 1.73	1.24 ± 0.87	4.37 ± 1.23	4.94 ± 0.60
HFD	29.68 ± 0.89	33.98 ± 1.94 ^a^	30.81 ± 1.57 ^a^	44.02 ± 2.74 ^a^	4.30 ± 1.97 ^a^	13.21 ± 2.72 ^a^	4.31 ± 0.73 ^b^
Reference							
Metformin	29.66 ± 1.49	33.93 ± 1.64 ^a^	30.79 ± 1.61 ^a^	36.68 ± 2.30 ^ac^	4.27 ± 0.91 ^a^	5.89 ± 2.05 ^c^	4.35 ± 0.64 ^b^
Test substances—WC							
200 mg/kg	29.02 ± 1.47	33.39 ± 1.38 ^a^	30.45 ± 1.26 ^a^	34.61 ± 1.85 ^bc^	4.37 ± 1.18 ^a^	4.16 ± 1.94 ^c^	4.31 ± 0.66 ^b^
100 mg/kg	29.13 ± 1.59	33.45 ± 1.59 ^a^	30.26 ± 1.85 ^a^	36.03 ± 2.73 ^ac^	4.32 ± 1.16 ^a^	5.77 ± 2.09 ^c^	4.31 ± 0.72 ^b^
50 mg/kg	29.23 ± 1.13	33.56 ± 1.41 ^a^	30.21 ± 1.79 ^a^	43.00 ± 2.41 ^a^	4.33 ± 0.62 ^a^	12.79 ± 2.69 ^a^	4.36 ± 0.64 ^b^

Values are expressed as mean ± SD of 10 mice. NFD = normal pellet diet; HFD = 45% Kcal high-fat diet; WC = water chestnut (fruit of *Trapa japonica* Flerov.) extracts; THSD = Tukey’s honest significant difference; NFD control = vehicle (distilled water) 10 mL/kg NFD orally administered to mice; metformin was administrated at a dose of 250 mg/kg; * all animals were overnight fasted; ^a^ *p* < 0.01 and ^b^ *p* < 0.05 as compared with NFD control by THSD test; ^c^ *p* < 0.01 as compared with HFD control by THSD test.

**Table 4:** Changes of absolute and relative organ weights in NFD- or HFD-supplied mice.

Organs Groups	Absolute Organ Weights (g)				
	Liver	Kidney	Pancreas	Periovarian Fat Pads	Abdominal Wall Fat Pads
Controls					
NFD	1.269 ± 0.064	0.195 ± 0.018	0.240 ± 0.045	0.072 ± 0.024	0.063 ± 0.030
HFD	1.887 ± 0.115 ^a^	0.289 ± 0.016 ^a^	0.232 ± 0.035	0.643 ± 0.116 ^c^	0.599 ± 0.080 ^a^
Reference					
Metformin	1.548 ± 0.103 ^ab^	0.239 ± 0.017 ^ab^	0.253 ± 0.033	0.302 ± 0.074 ^cd^	0.291 ± 0.062 ^ab^
Test materials—WC					
200 mg/kg	1.393 ± 0.152 ^b^	0.213 ± 0.016 ^b^	0.258 ± 0.042	0.219 ± 0.116 ^cd^	0.203 ± 0.080 ^ab^
100 mg/kg	1.548 ± 0.115 ^ab^	0.234 ± 0.018 ^ab^	0.250 ± 0.041	0.301 ± 0.053 ^cd^	0.293 ± 0.051 ^ab^
50 mg/kg	1.840 ± 0.139 ^a^	0.280 ± 0.023 ^ab^	0.245 ± 0.036	0.624 ± 0.119 ^c^	0.564 ± 0.099 ^a^
**Organs** **Groups**	**Relative Organ Weights (% of Body Weights)**				
	**Liver**	**Kidney**	**Pancreas**	**Periovarian** **Fat Pads**	**Abdominal wall** **Fat Pads**
Controls					
NFD	4.091 ± 0.299	0.628 ± 0.047	0.770 ± 0.129	0.229 ± 0.069	0.200 ± 0.092
HFD	4.294 ± 0.281	0.657 ± 0.034	0.526 ± 0.074 ^a^	1.466 ± 0.285 ^c^	1.362 ± 0.176 ^a^
Reference					
Metformin	4.230 ± 0.323	0.654 ± 0.063	0.690 ± 0.074 ^b^	0.826 ± 0.208 ^cd^	0.796 ± 0.167 ^ab^
Test materials–WC					
200 mg/kg	4.034 ± 0.482	0.618 ± 0.063	0.747 ± 0.117 ^b^	0.624 ± 0.302 ^cd^	0.581 ± 0.203 ^ab^
100 mg/kg	4.318 ± 0.462	0.653 ± 0.067	0.695 ± 0.112 ^b^	0.837 ± 0.143 ^cd^	0.817 ± 0.149 ^ab^
50 mg/kg	4.294 ± 0.441	0.653 ± 0.067	0.571 ± 0.090 ^a^	1.451 ± 0.274 ^c^	1.307 ± 0.187 ^a^

Values are expressed means ± SD of 10 mice. NFD = normal pellet diet; HFD = 45% Kcal high-fat diet; WC = water chestnut (fruit of *Trapa japonica* Flerov.) extracts; THSD = Tukey’s honest significant difference; MW = Mann–Whitney U; NFD control = vehicle (distilled water) 10 mL/kg NFD orally administered to mice; metformin was administrated at a dose level of 250 mg/kg; ^a^ *p* < 0.01 as compared with NFD control by THSD test; ^c^ *p* < 0.01 as compared with NFD control by MW test; ^b^ *p* < 0.01 as compared with HFD control by THSD test; ^d^ *p* < 0.01 as compared with HFD control by MW test.

**Table 5:** Changes on the Histopathology-Histomorphometry of the Periovarian and Abdominal Wall Deposited Fat Pads in NFD or HFD Supplied Mice.

Items Groups	Periovarian Fat Pads		Abdominal Wall Fat Pads	
	Thickness (mm)	Adipocyte Diameters (μm)	Thickness (mm)	Adipocyte Diameters (μm)
Controls				
NFD	1.43 ± 0.55	31.91 ± 6.49	1.17 ± 0.62	37.19 ± 6.42
HFD	5.81 ± 0.91 ^a^	99.26 ± 14.38 ^a^	6.77 ± 1.18 ^a^	131.31 ± 21.03 ^d^
Reference				
Metformin	3.34 ± 0.67 ^ac^	55.82 ± 12.68 ^ac^	3.81 ± 0.67 ^ac^	75.34 ± 11.65 ^de^
Test materials–WC				
200 mg/kg	2.65 ± 0.42 ^bc^	42.65 ± 10.41 ^c^	2.80 ± 0.67 ^ac^	59.45 ± 13.80 ^de^
100 mg/kg	3.35 ± 0.71 ^ac^	55.65 ± 11.59 ^ac^	3.83 ± 0.78 ^ac^	75.98 ± 14.69 ^de^
50 mg/kg	5.65 ± 1.46 ^a^	96.80 ± 20.55 ^a^	6.62 ± 1.51 ^a^	127.61 ± 22.50 ^d^

Values are expressed Means ± SD of 10 mice. NFD = normal pellet diet; HFD = 45% Kcal high-fat diet; WC = water chestnut (fruit of *Trapa japonica* Flerov.) extracts; THSD = Tukey’s honest significant difference; MW = Mann–Whitney U; NFD control = vehicle (distilled water) 10 mL/kg NFD orally administered to mice; metformin was administrated at a dose level of 250 mg/kg; ^a^ *p* < 0.01 and ^b^ *p* < 0.05 as compared with NFD control by THSD test; ^c^ *p* < 0.01 as compared with HFD control by THSD test; ^d^ *p* < 0.01 as compared with NFD control by MW test; ^e^ *p* < 0.01 as compared with HFD control by MW test.

**Table 6:** Changes on histopathology–histomorphometry of the pancreas in NFD- or HFD-supplied mice.

Items Groups	Zymogen Granules (%/mm^2^ of Exocrine)	Mean Islet Numbers (Numbers/10 mm^2^)	Mean Islet Diamete (μm/Islet)	Insulin-IR Cells (cells/mm^2^) (A)	Glucagon-IR Cells (cells/mm^2^) (B)	Insulin/Glucagon Ratio (A/B)
Controls						
NFD	57.80 ± 11.34	5.00 ± 1.49	111.04 ± 14.01	230.00 ± 50.24	71.80 ± 15.54	3.20 ± 0.16
HFD	11.46 ± 3.34 ^c^	20.00 ± 1.89 ^c^	228.10 ± 30.15 ^c^	2801.60 ± 270.08 ^c^	439.40 ± 59.40 ^a^	6.43 ± 0.62 ^c^
Reference						
Metformin	27.37 ± 4.88 ^cd^	11.80 ± 2.90 ^cd^	168.55 ± 15.66 ^cd^	1207.50 ± 261.04 ^cd^	288.10 ± 64.21 ^ab^	4.20 ± 0.12 ^cd^
Test materials—WC						
200 mg/kg	38.76 ± 7.97 ^cd^	8.10 ± 1.52 ^cd^	148.70 ± 14.65 ^cd^	737.70 ± 157.23 ^cd^	204.90 ± 47.80 ^ab^	3.61 ± 0.10 ^cd^
100 mg/kg	27.13 ± 6.38 ^cd^	11.90 ± 2.88 ^cd^	168.42 ± 12.47 ^cd^	1243.40 ± 252.14 ^cd^	289.40 ± 55.85 ^ab^	4.29 ± 0.13 ^cd^
50 mg/kg	11.75 ± 4.91 ^c^	19.20 ± 3.39 ^c^	222.91 ± 37.97 ^c^	2728.10 ± 532.60 ^c^	429.10 ± 85.38 ^a^	6.36 ± 0.19 ^c^

Values are expressed means ± SD of 10 mice. NFD = normal pellet diet; HFD = 45% Kcal high-fat diet; WC = water chestnut (fruit of *Trapa japonica* Flerov.) extracts; THSD = Tukey’s honest significant difference; MW = Mann–Whitney U; NFD control = vehicle (distilled water) 10 mL/kg NFD orally administered to mice; metformin was administrated at a dose level of 250 mg/kg; ^a^ *p* < 0.01 and ^b^ *p* < 0.05 as compared with intact control by THSD test; ^d^ *p* < 0.01 as compared with intact control by MW test; ^c^ *p* < 0.01 as compared with HFD control by THSD test-.

**Table 7:** Changes in blood glucose levels and serum lipid contents in HFD- or NFD-supplied mice.

Items Groups	Glucose (mg/dL)	Total Cholesterol (mg/dL)	Triglyceride (mg/dL)	Low-Density Lipoprotein (mg/dL)	High-Density Lipoprotein (mg/dL)
Controls					
NFD	90.90 ± 11.82	89.30 ± 12.01	74.50 ± 10.46	17.40 ± 3.50	94.80 ± 17.84
HFD	258.70 ± 57.74 ^d^	278.40 ± 31.21 ^d^	252.50 ± 53.96 ^a^	87.10 ± 16.82 ^a^	23.30 ± 6.04 ^a^
Reference					
Metformin	141.70 ± 15.41 ^de^	162.40 ± 24.97 ^de^	145.90 ± 17.63 ^ac^	50.30 ± 14.01 ^ac^	49.50 ± 10.37 ^ac^
Test materials—WC					
200 mg/kg	111.30 ± 17.54 ^de^	123.30 ± 17.96 ^de^	112.80 ± 30.68 ^c^	38.80 ± 14.16 ^bc^	59.40 ± 17.72 ^ac^
100 mg/kg	140.40 ± 22.02 ^de^	161.30 ± 24.45 ^de^	145.70 ± 26.38 ^ac^	49.90 ± 11.26 ^ac^	49.70 ± 13.22 ^ac^
50 mg/kg	252.70 ± 65.34 ^d^	268.40 ± 46.15 ^d^	247.40 ± 59.53 ^a^	83.10 ± 22.99 ^a^	24.10 ± 11.62 ^a^

Values are expressed means ± SD of 10 mice. NFD = normal pellet diet; HFD = 45% Kcal high-fat diet; WC = water chestnut (fruit of *Trapa japonica* Flerov.) extracts; THSD = Tukey’s honest significant difference; MW = Mann–Whitney U; NFD control = vehicle (distilled water) 10 mL/kg NFD orally administered to mice. Metformin was administrated at a dose level of 250 mg/kg; ^a^ *p* < 0.01 and ^b^ *p* < 0.05 as compared with NFD control by THSD test; ^d^ *p* < 0.01 as compared with NFD control by MW test; ^c^ *p* < 0.01 as compared with HFD control by THSD test; ^e^ *p* < 0.01 as compared with HFD control by MW test.

**Table 8:** Changes of serum AST, ALT, ALP, LDH, GGT, BUN, and creatine levels in HFD- or NFD-administered mice.

Items Groups	AST (IU/L)	ALT (IU/L)	ALP (IU/L)	LDH (×10 IU/L)	GGT (IU/L)	BUN (mg/dL)	Creatinine (mg/dL)
Controls							
NFD	82.20 ± 14.51	45.60 ± 14.51	73.00 ± 15.04	51.62 ± 14.19	4.30 ± 2.21	31.80 ± 11.68	0.60 ± 0.16
HFD	219.40 ± 40.68 ^d^	155.90 ± 14.98 ^a^	214.60 ± 46.61 ^d^	404.42 ± 107.31 ^d^	16.40 ± 2.84 ^a^	111.00 ± 18.15 ^d^	2.14 ± 0.40 ^a^
Reference							
Metformin	139.90 ± 14.22 ^df^	98.80 ± 16.14 ^ac^	138.60 ± 16.75 ^df^	224.78 ± 26.41 ^df^	9.10 ± 1.60 ^ac^	61.90 ± 16.38 ^df^	1.18 ± 0.27 ^ac^
Test materials–WC							
200 mg/kg	105.40 ± 19.47 ^ef^	74.80 ± 16.00 ^bc^	101.80 ± 19.54 ^df^	163.52 ± 39.41 ^df^	6.60 ± 2.01 ^c^	45.80 ± 8.90 ^df^	0.87 ± 0.23 ^c^
100 mg/kg	138.60 ± 20.42 ^df^	96.00 ± 21.52 ^ac^	139.30 ± 13.74 ^df^	221.25 ± 30.55 ^df^	9.10 ± 1.91 ^ac^	62.30 ± 14.53 ^df^	1.17 ± 0.31 ^ac^
50 mg/kg	215.70 ± 52.90 ^d^	151.20 ± 27.39 ^a^	209.30 ± 48.63 ^d^	383.11 ± 123.57 ^d^	16.00 ± 4.24 ^a^	107.80 ± 27.82 ^d^	2.07 ± 0.53 ^a^

Values are expressed means ± SD of 10 mice; HFD = 45% Kcal high-fat diet; NFD = normal pellet diet; WC = water chestnut (fruit of *Trapa japonica* Flerov.) extracts; THSD = Tukey’s honest significant difference; MW = Mann–Whitney U. Full name of serum biochemistry items are listed in Table 3. NFD control = vehicle (distilled water) 10 mL/kg NFD orally administered to mice; metformin was administrated at a dose level of 250 mg/kg; ^a^ *p* < 0.01 and ^b^ *p* < 0.05 as compared with NFD control by THSD test; ^d^ *p* < 0.01 and ^e^ *p* < 0.05 as compared with NFD control by MW test; ^c^ *p* < 0.01 as compared with HFD control by THSD test; ^f^ *p* < 0.01 as compared with HFD control by MW test.

**Table 9:** Changes in histopathology–histomorphometry of the liver and kidney in HFD- or NFD-administered mice.

Items Groups	Liver Steatosis (%/mm^2^ of Hepatic Tissues)	Mean Hepatocyte Diameters (μm/cell)	Degenerative Renal Tubule Numbers (%)
Controls			
NFD	9.55 ± 4.68	15.93 ± 2.70	4.40 ± 2.91
HFD	80.77 ± 10.42 ^a^	42.29 ± 8.12 ^a^	75.70 ± 10.27 ^a^
Reference			
Metformin	44.57 ± 10.20 ^ab^	23.61 ± 3.43 ^ab^	41.30 ± 10.32 ^ab^
Test materials–WC			
200 mg/kg	28.17 ± 10.74 ^ab^	19.97 ± 1.51 ^ab^	16.90 ± 5.57 ^ab^
100 mg/kg	44.59 ± 12.01 ^ab^	23.82 ± 2.95 ^ab^	41.60 ± 11.57 ^ab^
50 mg/kg	78.11 ± 13.04 ^a^	41.04 ± 8.82 ^a^	73.80 ± 15.27 ^a^

Values are expressed means ± SD of 10 mice; NFD = normal pellet diet; HFD = 45% Kcal high-fat diet; WC = water chestnut (fruit of *Trapa japonica* Flerov.) extracts; MW = Mann–Whitney U; NFD control = vehicle (distilled water) 10 mL/kg NFD orally administered to mice; metformin was administrated at a dose level of 250 mg/kg; ^a^ *p* < 0.01 as compared with NFD control by MW test ^b^ *p* < 0.01 as compared with HFD control by MW test.

**Table 10:** Changes in the liver lipid peroxidation and antioxidant defense systems in HFD- or NFD-administered mice.

Items Groups	Lipid Peroxidation	Antioxidant Defense System		
	Malondialdehyde (nM/mg Tissue)	Glutathione (μM/mg Tissue)	Catalase (U/mg Tissue)	SOD (U/mg Tissue)
Controls				
NFD	9.34 ± 1.84	69.63 ± 10.20	71.24 ± 12.12	7.84 ± 1.21
HFD	78.37 ± 11.04 ^a^	10.45 ± 1.80 ^a^	10.32 ± 2.61 ^a^	0.78 ± 0.26 ^a^
Reference				
Metformin	45.07 ± 11.48 ^ab^	32.31 ± 10.11 ^ab^	31.81 ± 10.21 ^ab^	2.39 ± 0.46 ^ab^
Test materials–WC				
200 mg/kg	34.30 ± 11.03 ^ab^	45.49 ± 13.68 ^ab^	44.43 ± 10.39 ^ab^	3.36 ± 1.11 ^ab^
100 mg/kg	45.99 ± 10.48 ^ab^	32.31 ± 13.52 ^ab^	31.42 ± 10.45 ^ab^	2.40 ± 0.58 ^ab^
50 mg/kg	76.28 ± 16.31 ^a^	10.74 ± 3.21 ^a^	11.04 ± 3.44 ^a^	0.82 ± 0.40 ^a^

Values are expressed means ± SD of 10 mice; NFD = normal pellet diet; HFD = 45% Kcal high-fat diet; WC = water chestnut (fruit of *Trapa japonica* Flerov.) extracts; SOD = superoxide dismutase; MW = Mann–Whitney U; NFD control = vehicle (distilled water) 10 mL/kg NFD orally administered to mice. Metformin was administrated at a dose level of 250 mg/kg; ^a^ *p* < 0.01 as compared with NFD control by MW test; ^b^ *p* < 0.01 as compared with HFD control by MW test.

**Table 11:** Changes in the hepatic glucose-regulating enzyme activities in NFD- or HFD-administered mice.

Items Groups	Glucokinase (nM/min/mg Protein)	Glucose-6-phosphatase (nM/min/mg Protein)	PEPCK (nM/min/mg Protein)
Controls			
NFD	5.95 ± 1.19	110.04 ± 25.44	1.56 ± 0.59
HFD	1.64 ± 0.43 ^c^	309.59 ± 57.05 ^c^	5.46 ± 0.79 ^a^
Reference			
Metformin	3.14 ± 0.49 ^cd^	173.99 ± 22.39 ^cd^	3.20 ± 0.77 ^ab^
Test materials–WC			
200 mg/kg	4.11 ± 0.75 ^cd^	145.77 ± 18.21 ^cd^	2.39 ± 0.60 ^b^
100 mg/kg	3.14 ± 0.45 ^cd^	174.79 ± 22.81 ^cd^	3.20 ± 0.71 ^ab^
50 mg/kg	1.69 ± 0.46 ^c^	299.60 ± 80.39 ^c^	5.33 ± 1.33 ^a^

Values are expressed means ± SD of 10 mice; NFD = normal pellet diet; HFD = 45% Kcal high-fat diet; WC = water chestnut (fruit of *Trapa japonica* Flerov.) extracts; PEPCK = phosphoenolpyruvate carboxykinase; THSD = Tukey’s honest significant difference; MW = Mann–Whitney U; NFD control = vehicle (distilled water) 10 mL/kg NFD orally administered to mice. Metformin was administrated at a dose level of 250 mg/kg; ^a^ *p* < 0.01 as compared with NFD control by THSD test; ^b^ *p* < 0.01 as compared with HFD control by THSD test; ^c^ *p* < 0.01 as compared with NFD control by MW test; ^d^ *p* < 0.01 as compared with HFD control by MW test.

**Table 12:** Changes of lipid metabolism-related gene mRNA expressions in liver of NFD- or HFD-supplied mice in NFD- or HFD-administered mice.

Items Groups	Hepatic Tissue (Relative to Control/GAPDH)		
	ACC1	AMPKα1	AMPKα2
Controls			
NFD	1.00 ± 0.07	1.01 ± 0.12	1.00 ± 0.07
HFD	5.31 ± 1.01 ^c^	0.27 ± 0.07 ^a^	0.25 ± 0.05 ^c^
Reference			
Metformin	3.10 ± 0.77 ^cd^	0.48 ± 0.08 ^ab^	0.47 ± 0.12 ^cd^
Test materials–WC			
200 mg/kg	2.30 ± 0.46 ^cd^	0.60 ± 0.12 ^ab^	0.70 ± 0.16 ^cd^
100 mg/kg	3.09 ± 0.40 ^cd^	0.48 ± 0.11 ^ab^	0.47 ± 0.09 ^cd^
50 mg/kg	5.16 ± 1.02 ^c^	0.28 ± 0.11 ^a^	0.27 ± 0.10 ^c^

Values are expressed means ± SD of 10 mice; NFD = normal pellet diet; HFD = 45% Kcal high-fat diet; WC = water chestnut (fruit of *Trapa japonica* Flerov.) extracts; GAPDH = glyceraldehyde 3-phosphate dehydrogenase; THSD = Tukey’s honest significant difference; MW = Mann–Whitney U; full name and sequence of genes are listed in Table 4; NFD control = vehicle (distilled water) 10 mL/kg NFD orally administered to mice; Metformin was administrated at a dose level of 250 mg/kg; ^a^ *p* < 0.01 as compared with NFD control by THSD test; ^b^ *p* < 0.01 as compared with HFD control by THSD test; ^c^ *p* < 0.01 as compared with NFD control by MW test; ^d^ *p* < 0.01 as compared with HFD control by MW test.

**Table 13:** Changes in lipid metabolism-related gene mRNA expressions in adipose tissue of NFD- or HFD-administered mice, real-time RT-PCR Analysis.

Groups Items	Control		Reference	Test Materials—WC		
	NFD	HFD	Metformin	200 mg/kg	100 mg/kg	50 mg/kg
Adipose tissue (Relative to control/GAPDH)						
Leptin	1.00 ± 0.06	8.57 ± 1.27 ^c^	4.68 ± 1.13 ^cd^	3.44 ± 0.83 ^cd^	4.79 ± 1.19 ^cd^	8.18 ± 2.42 ^c^
UCP2	1.01 ± 0.09	0.24 ± 0.05 ^a^	0.47 ± 0.13 ^ab^	0.63 ± 0.15 ^ab^	0.47 ± 0.13 ^ab^	0.24 ± 0.08 ^a^
Adiponectin	1.00 ± 0.08	0.15 ± 0.09 ^a^	0.40 ± 0.09 ^ab^	0.59 ± 0.14 ^ab^	0.41 ± 0.08 ^ab^	0.16 ± 0.09 ^a^
C/EBPα	1.00 ± 0.07	3.14 ± 0.66 ^c^	1.84 ± 0.24 ^cd^	1.33 ± 0.29 ^cd^	1.81 ± 0.28 ^cd^	3.08 ± 1.20 ^c^
C/EBPβ	1.00 ± 0.07	4.11 ± 0.68 ^c^	2.41 ± 0.51 ^cd^	1.84 ± 0.28 ^cd^	2.41 ± 0.51 ^cd^	4.00 ± 0.93 ^c^
SREBP1 c	1.00 ± 0.06	2.97 ± 0.86 ^c^	1.73 ± 0.20 ^cd^	1.27 ± 0.15 ^cd^	1.73 ± 0.29 ^cd^	2.90 ± 0.99 ^c^
PPARα	1.00 ± 0.09	0.16 ± 0.05 ^c^	0.32 ± 0.06 ^cd^	0.50 ± 0.18 ^cd^	0.31 ± 0.08 ^cd^	0.17 ± 0.06 ^c^
PPARγ	1.01 ± 0.10	8.09 ± 0.91 ^c^	4.63 ± 0.97 ^cd^	3.61 ± 1.07 ^cd^	4.68 ± 1.22 ^cd^	7.82 ± 1.38 ^c^
FAS	1.00 ± 0.05	15.40 ± 3.36 ^c^	8.86 ± 0.63 ^cd^	6.91 ± 1.85 ^cd^	8.85 ± 0.85 ^cd^	15.06 ± 4.30 ^c^

Values are expressed as mean ± SD of 10 mice; NFD = normal pellet diet; HFD = 45% Kcal high-fat diet; WC = water chestnut (fruit of *Trapa japonica* Flerov.) extracts; GAPDH = glyceraldehyde 3-phosphate dehydrogenase; THSD = Tukey’s honest significant difference; MW = Mann–Whitney U; NFD control = vehicle (distilled water) 10 mL/kg NFD orally administered to mice; metformin was administrated at a dose level of 250 mg/kg; ^a^ *p* < 0.01 as compared with NFD control by THSD test; ^c^ *p* < 0.01 as compared with NFD control by MW test; ^b^ *p* < 0.01 as compared with HFD control by THSD test; ^d^ *p* < 0.01 as compared with HFD control by MW test.

## Data Availability

Data supporting reported results are available upon request.

## References

[1] Katsiki N., Athyros V.G., Karagiannis A., Mikhailidis D.P. (2014). Characteristics other than the diagnostic criteria associated with metabolic syndrome: An overview. Curr. Vasc. Pharmacol..

[2] Wendel A.A., Purushotham A., Liu L.F., Belury M.A. (2008). Conjugated linoleic acid fails to worsen insulin resistance but induces hepatic steatosis in the presence of leptin in ob/ob mice. J. Lipid Res..

[3] Tilg H., Moschen A.R. (2006). Adipocytokines: Mediators linking adipose tissue, inflammation and immunity. Nat. Rev. Immunol..

[4] James P.T., Leach R., Kalamara E., Shayeghi M. (2001). The worldwide obesity epidemic. Obes. Res..

[5] World Health Organization Diabetes. https://www.who.int/news-room/fact-sheets/detail/diabetes.

[6] Lebovitz H.E. (2001). Insulin resistance: Definition and consequences. Exp. Clin. Endocrinol. Diabetes.

[7] Goldstein B.J. (2002). Insulin resistance as the core defect in type 2 diabetes mellitus. Am. J. Cardiol..

[8] Angulo P. (2002). Nonalcoholic fatty liver disease. N. Engl. J. Med..

[9] Kadowaki T., Yamauchi T. (2005). Adiponectin and adiponectin receptors. Endocr. Rev..

[10] Yun S.N., Moon S.J., Ko S.K., Im B.O., Chung S.H. (2004). Wild ginseng prevents the onset of high-fat diet induced hyperglycemia and obesity in ICR mice. Arch. Pharm. Res..

[11] Kim J.W., Lee Y.S., Seol D.J., Cho I.J., Ku S.K., Choi J.S., Lee H.J. (2018). Anti-obesity and fatty liver-preventing activities of *Lonicera caerulea* in high-fat diet-fed mice. Int. J. Mol. Med..

[12] Choi B.R., Kim H.J., Lee Y.J., Ku S.K. (2020). Anti-diabetic obesity effects of *Wasabia japonica* Matsum leaf extract on 45% Kcal high-fat diet-fed mice. Nutrients.

[13] Choi E.H., Chun Y.S., Kim J.K., Ku S.K., Jeon S.W., Park T.S., Shim S.M. (2020). Modulating lipid and glucose metabolism by glycosylated kaempferol rich roasted leaves of *Lycium chinense* via upregulating adiponectin and AMPK activation in obese mice-induced type 2 diabetes. J. Funct. Foods.

[14] Sone H., Suzuki H., Takahashi A., Yamada N. (2001). Disease model: Hyperinsulinemia and insulin resistance. Part A-targeted disruption of insulin signaling or glucose transport. Trends Mol. Med..

[15] Ku S.K., Sung S.H., Choung J.J., Choi J.-S., Shin Y.K., Kim J.W. (2016). Anti-obesity and anti-diabetic effects of a standardized potato extract in ob/ob mice. Exp. Ther. Med..

[16] Inzucchi S.E. (2002). Oral antihyperglycemic therapy for type 2 diabetes: Scientific review. JAMA.

[17] Chen H., Quz Z., Fu L., Dong P., Zhang X. (2009). Physicochemical properties and antioxidant capacity of 3 polysaccharides from green tea, oolong tea, and black tea. J. Food Sci..

[18] Hays N.P., Galassetti P.R., Coker R.H. (2008). Prevention and treatment of type 2 diabetes: Current role of lifestyle, natural product, and pharmacological interventions. Pharmacol. Ther..

[19] Kwon Y.I., Apostolidis E., Shetty K. (2008). In vitro studies of eggplant (*Solanum melongena*) phenolics as inhibitors of key enzymes relevant for type 2 diabetes and hypertension. Bioresour. Technol..

[20] Ma A., Wang J., Yang L., An Y., Zhu H. (2017). AMPK activation enhances the anti-atherogenic effects of high density lipoproteins in apoE(−/−) mice. J. Lipid Res..

[21] Mottillo E.P., Desjardins E.M., Fritzen A.M., Zou V.Z., Crane J.D., Yabut J.M., Kiens B., Erion D.M., Lanba A., Granneman J.G. (2017). FGF21 does not require adipocyte AMP-activated protein kinase (AMPK) or the phosphorylation of acetyl-CoA carboxylase (ACC) to mediate improvements in whole-body glucose homeostasis. Mol. Metab..

[22] Davidson M.B., Peters A.L. (1997). An overview of metformin in the treatment of type 2 diabetes mellitus. Am. J. Med..

[23] Jang J.D., Kim M., Nam G.H., Kim Y.M., Kang S.M., Lee K.Y., Park Y.J. (2020). Antiaging activity of peptide identified from fermented *Trapa Japonica* fruit extract in human dermal fibroblasts. Evid. Based Complement. Altern. Med..

[24] Yasuda M., Yasutake K., Hino M., Ohwatari H., Ohmagari N., Takedomi K., Tanaka T., Nonaka G. (2014). Inhibitory effects of polyphenols from water chestnut (*Trapa japonica*) husk on glycolytic enzymes and postprandial blood glucose elevation in mice. Food Chem..

[25] Kim B., Kim J.E., Choi B.K., Kim H.S. (2015). Anti-Inflammatory Effects of water chestnut extract on cytokine responses via nuclear factor-κB-signaling pathway. Biomol. Ther..

[26] Kim Y.S., Hwang J.W., Jang J.H., Son S., Seo I.B., Jeong J.H., Kim E.H., Moon S.H., Jeon B.T., Park P.J. (2016). *Trapa japonica* pericarp extract reduces LPS-induced inflammation in macrophages and acute lung injury in mice. Molecules.

[27] Wang C.C.R., Ciou J.Y., Chiang P.Y. (2009). Effect of micronization on functional properties of the water caltrop (*Trapa taiwanensis* Nakai) pericarp. Food Chem..

[28] Shindo K., Kuroki E., Toyoda M. (2013). Antioxidative compounds contained in the seed with hard shell of *Trapa japonica* Flerov. and its herbal tea. J. Home Econ. Jpn..

[29] Lee D., Lee O.H., Choi G., Kim J.D. (2017). Antioxidant and anti-adipogenic activities of *Trapa japonica* shell extract cultivated in Korea. Prev. Nutr. Food Sci..

[30] Park C., Hwang Y., Hwang B.S., Shin S.Y., Cho P.Y., Lee S.Y., Choi K.M., Lee K.W., Kim G.Y., Cho Y.H. (2019). *Trapa japonica* inhibits adipocyte differentiation and adipogenesis through AMPK signaling pathway in 3T3-L1 pre-adipocytes. Int. J. Food Sci. Nutr..

[31] Kang M.J., Lee S.K., Song J.H., Kim M.E., Kim M.J., Jang J.S., Lee J.H., Kim J.I. (2009). Water chestnut (*Trapa japonica* Flerov.) exerts inhibitory effect on postprandial glycemic response in rats and free radical scavenging activity in vitro. Food Sci. Biotechnol..

[32] Seufert J., Lubben G., Dietrich K., Bates P.C. (2004). A comparison of the effects of thiazolidinediones and metformin on metabolic control in patients with type 2 diabetes mellitus. Clin. Ther..

[33] Park S.H., Ko S.K., Chung S.H. (2005). *Euonymus alatus* prevents the hyperglycemia and hyperlipidemia induced by high-fat diet in ICR mice. J. Ethnopharmacol..

[34] Lim J.M., Song C.-H., Park S.-J., Park D.-C., Cho H.-R., Jung G.-W., Bashir K.M.I., Ku S.K., Choi J.-S. (2018). Protective effects of triple fermented barley extracts (FBe) on HCl/EtOH-induced gastric mucosa damages in mice. Food Sci. Nutr..

[35] Morton D.B., Griffiths P.H. (1985). Guidelines on the recognition of pain, distress and discomfort in experimental animals and an hypothesis for assessment. Vet. Rec..

[36] Flecknell P. (1996). Laboratory Animal Anesthesia.

[37] Korea Food and Drug Administration (2017). Testing Guidelines for Safety Evaluation of Drugs.

[38] Folch J., Lees M., Sloane-Stanley G.H. (1957). A simple method for the isolation and purification of total lipids from animal tissues. J. Biol. Chem..

[39] Kavutcu M., Canbolat O., Oztürk S., Olcay E., Ulutepe S., Ekinci C., Gökhun I.H., Durak I. (1996). Reduced enzymatic antioxidant defense mechanism in kidney tissues from gentamicin-treated guinea pigs: Effects of vitamins E and C. Nephron.

[40] Jamall I.S., Smith J.C. (1985). Effects of cadmium on glutathione peroxidase, superoxidase dismutase and lipid peroxidation in the rat heart: A possible mechanism of cadmium cardiotoxicity. Toxicol. Appl. Pharmacol..

[41] Lowry O.H., Rosenbrough N.J., Farr A.L., Randall R.J. (1951). Protein measurement with the Folin phenol reagent. J. Biol. Chem..

[42] Sedlak J., Lindsay R.H. (1968). Estimation of total, protein-bound, and nonprotein sulfhydryl groups in tissue with Ellman’s reagent. Anal. Biochem..

[43] Aebi H., Bergmeyer H.U. (1974). Catalase. Methods in Enzymatic Analysis.

[44] Sun Y., Larry W.O., Ying L.A. (1988). Simple method for clinical assay of superoxide dismutase. Clin. Chem..

[45] Hulcher F.H., Oleson W.H. (1973). Simplified spectrophotometric assay for microsomal 3-hydroxy-3-methylglutaryl CoA reductase by measurement of coenzyme A. J. Lipid Res..

[46] Davidson A.L., Arion W.J. (1987). Factors underlying significant underestimations of glucokinase activity in crude liver extracts: Physiological implications of higher cellular activity. Arch. Biochem. Biophys..

[47] Alegre M., Ciudad C.J., Fillat C., Guinovart J.J. (1988). Determination of glucose-6-phosphatase activity using the glucose dehydrogenase-coupled reaction. Anal. Biochem..

[48] Bentle L.A., Lardy H.A. (1976). Interaction of anions and divalent metal ions with phosphoenolpyruvate carboxykinase. J. Biol. Chem..

[49] Veiga F.M.S., Graus-Nunes F., Rachid T.L., Barreto A.B., Mandarim-de-Lacerda C.A., Souza-Mello V. (2017). Anti-obesogenic effects of WY14643 (PPAR-alpha agonist): Hepatic mitochondrial enhancement and suppressed lipogenic pathway in diet-induced obese mice. Biochimie.

[50] Sung Y.Y., Kim D.S., Kim S.H., Kim H.K. (2017). Anti-obesity activity, acute toxicity, and chemical constituents of aqueous and ethanol Viola mandshurica extracts. BMC Complement. Altern. Med..

[51] Schmittgen T.D., Livak K.J. (2008). Analyzing real-time PCR data by the comparative C_T_ method. Nat. Protoc..

[52] Levene A. (1981). Pathological factors influencing excision of tumours in the head and neck. Part I. Clin. Otolaryngol. Allied Sci..

[53] Williams C.D., Stengel J., Asike M.I., Torres D.M., Shaw J., Contreras M., Landt C.L., Harrison S.A. (2011). Prevalence of nonalcoholic fatty liver disease and nonalcoholic steatohepatitis among a largely middleaged population utilizing ultrasound and liver biopsy: A prospective study. Gastroenterology.

[54] Tan Y., Kim J., Cheng J., Ong M., Lao W.G., Jin X.L., Lin Y.G., Xiao L., Zhu X.Q., Qu X.Q. (2017). Green tea polyphenols ameliorate non-alcoholic fatty liver disease through upregulating AMPK activation in high fat fed Zucker fatty rats. World J. Gastroenterol..

[55] Pais R., Charlotte F., Fedchuk L., Bedossa P., Lebray P., Poynard T., Ratziu V. (2013). A systematic review of follow-up biopsies reveals disease progression in patients with non-alcoholic fatty liver. J. Hepatol..

[56] Rotman Y., Sanyal A.J. (2017). Current and upcoming pharmacotherapy for non-alcoholic fatty liver disease. Gut.

[57] Gartner L.P., Hiatt J.L. (2007). Color Textbook of Histology.

[58] Wilson J.S., Korsten M.A., Leo M.A., Lieber C.S. (1988). Combined effects of protein deficiency and chronic ethanol consumption on rat pancreas. Dig. Dis. Sci..

[59] Bookchin R.M., Gallop P.M. (1968). Structure of hemoglobin AIc; nature of the N-terminal beta chain blocking group. Biochem. Biophys. Res. Commun..

[60] Larsen M.L., Hørder M., Mogensen E.F. (1990). Effect of long-term monitoring of glycosylated hemoglobin levels in insulin-dependent diabetes mellitus. N. Engl. J. Med..

[61] Sodikoff C.H. (1995). Laboratory Profiles of Small Animal Diseases: A Guide to Laboratory Diagnosis.

[62] Garg M.C., Singh K.P., Bansal D.D. (1997). Effect of vitamin C supplementation on oxidative stress in experimental diabetes. Indian J. Exp. Biol..

[63] Giugliano D., Ceriello A., Paolisso G. (1996). Oxidative stress and diabetic vascular complications. Diabetes Care.

[64] Comporti M. (1985). Lipid peroxidation and cellular damage in toxic liver injury. Lab. Investig..

[65] Odabasoglu F., Cakir A., Suleyman H., Aslan A., Bayir Y., Halici M., Kazaz C. (2006). Gastroprotective and antioxidant effects of usnic acid on indomethacin-induced gastric ulcer in rats. J. Ethnopharmacol..

[66] Cheeseman K.H., Slater T.F. (1993). An introduction to free radical biochemistry. Br. Med. Bull..

[67] Erejuwa O.O., Sulaiman S.A., Wahab M.S., Salam S.K., Salleh M.S., Gurtu S. (2011). Comparison of antioxidant effects of honey, glibenclamide, metformin, and their combinations in the kidneys of streptozotocin-induced diabetic rats. Int. J. Mol. Sci..

[68] Chung S.I., Rico C.W., Kang M.Y. (2014). Comparative study on the hypoglycemic and antioxidative effects of fermented paste (doenjang) prepared from soybean and brown rice mixed with rice bran or red ginseng marc in mice fed with high fat diet. Nutrients.

[69] Wu D., Zheng N., Qi K., Cheng H., Sun Z., Gao B., Zhang Y., Pang W., Huangfu C., Ji S. (2015). Exogenous hydrogen sulfide mitigates the fatty liver in obese mice through improving lipid metabolism and antioxidant potential. Med. Gas Res..

[70] Ferre T., Riu E., Bosch F., Valera A. (1996). Evidence from transgenic mice that glucokinase is rate limiting for glucose utilization in the liver. FASEB J..

[71] Coope G.J., Atkinson A.M., Allott C., McKerrecher D., Johnstone C., Pike K.G., Holme P.C., Vertigan H., Gill D., Coghlan M.P. (2006). Predictive blood glucose lowering efficacy by Glucokinase activators in high fat fed female Zucker rats. Br. J. Pharmacol..

[72] She P., Shiota M., Shelton K.D., Chalkley R., Postic C., Magnuson M.A. (2000). Phosphoenolpyruvate carboxykinase is necessary for the integration of hepatic energy metabolism. Mol. Cell. Biol..

[73] Van Schaftingen E., Gerin I. (2002). The glucose-6-phosphatase system. Biochem. J..

[74] Zhou G., Myers R., Li Y., Chen Y., Shen X., Fenyk-Melody J., Wu M., Ventre J., Doebber T., Fujii N. (2001). Role of AMP-activated protein kinase in mechanism of metformin action. J. Clin. Investig..

[75] Lin C.H., Kuo Y.H., Shih C.C. (2014). Effects of Bofu-Tsusho-San on diabetes and hyperlipidemia associated with AMP-activated protein kinase and glucose transporter 4 in high-fat-fed mice. Int. J. Mol. Sci..

[76] Malini P., Kanchana G., Rajadurai M.U.R.U. (2011). Antidiabetic efficacy of ellagic acid in streptozotocin-induced diabetes mellitus in albino wistar rats. Asian J. Pharm. Clin. Res..

[77] Ahad A., Ganai A.A., Mujeeb M., Siddiqui W.A. (2014). Ellagic acid, an NF-κB inhibitor, ameliorates renal function in experimental diabetic nephropathy. Chem.-Biol. Interact..

[78] Farbood Y., Rashno M., Ghaderi S., Khoshnam S.E., Sarkaki A., Rashidi K., Rashno M., Badavi M. (2019). Ellagic acid protects against diabetes-associated behavioral deficits in rats: Possible involved mechanisms. Life Sci..

